# *ERBB2* mutation is associated with sustained tumor cell proliferation after short-term preoperative endocrine therapy in early lobular breast cancer

**DOI:** 10.1038/s41379-022-01130-7

**Published:** 2022-07-16

**Authors:** Isabel Grote, Stephan Bartels, Henriette Christgen, Martin Radner, Malte Gronewold, Leonie Kandt, Mieke Raap, Ulrich Lehmann, Oleg Gluz, Monika Graeser, Sherko Kuemmel, Ulrike Nitz, Nadia Harbeck, Hans Kreipe, Matthias Christgen

**Affiliations:** 1 Institute of Pathology, Hannover Medical School, Hannover, Germany; 2 West German Study Group, Moenchengladbach, Germany; 3 Ev. Bethesda Hospital, Moenchengladbach, Germany; 4 University Clinics Cologne, Women’s Clinic and Breast Center, Cologne, Germany; 5 University Medical Center Hamburg, Department of Gynecology, Hamburg, Germany; 6 Clinics Essen-Mitte, Breast Unit, Essen, Germany; 7 Charité, Women’s Clinic, Berlin, Germany; 8 LMU University Hospital, Breast Center, Department OB&GYN and CCC Munich, Munich, Germany

**Keywords:** Breast cancer, Medical research

## Abstract

Invasive lobular breast cancer (ILC) is a special breast cancer (BC) subtype and is mostly hormone receptor (HR)-positive and *ERBB2* non-amplified. Endocrine therapy restrains tumor proliferation and is the mainstay of lobular BC treatment. Mutation of *ERBB2* has been associated with recurrent ILC. However, it is unknown whether *ERBB2* mutation impacts on the otherwise exquisite responsiveness of early ILC to endocrine therapy. We have recently profiled *n* = 622 HR-positive early BCs from the ADAPT trial for mutations in candidate genes involved in endocrine resistance, including *ERBB2*. All patients were treated with short-term preoperative endocrine therapy (pET, tamoxifen or aromatase inhibitors) before tumor resection. Tumor proliferation after endocrine therapy (post-pET Ki67 index) was determined prospectively by standardized central pathology assessment supported by computer-assisted image analysis. Sustained or suppressed proliferation were defined as post-pET Ki67 ≥10% or <10%. Here, we report a subgroup analysis pertaining to ILCs in this cohort. ILCs accounted for 179/622 (28.8%) cases. ILCs were enriched in mutations in *CDH1* (124/179, 69.3%, *P* < 0.0001) and *ERBB2* (14/179, 7.8%, *P* < 0.0001), but showed fewer mutations in *TP53* (7/179, 3.9%, *P* = 0.0048) and *GATA3* (11/179, 6.1%, *P* < 0.0001). Considering all BCs irrespective of subtypes, *ERBB2* mutation was not associated with proliferation. In ILCs, however, *ERBB2* mutations were 3.5-fold more common in cases with sustained post-pET proliferation compared to cases with suppressed post-pET proliferation (10/75, 13.3% versus 4/104, 3.8%, *P* = 0.0248). Moreover, *ERBB2* mutation was associated with high Oncotype DX recurrence scores (*P* = 0.0087). In summary, our findings support that *ERBB2* mutation influences endocrine responsiveness in early lobular BC.

## Introduction

Invasive lobular breast cancer (ILC) is a special histological BC subtype^1,2^. Lobular BC is typically hormone receptor (HR)-positive and HER2/*ERBB2* non-amplified^1^. Lobular BC is driven by mutational inactivation of *CDH1*/E-cadherin and is a slow-growing tumor entity^3,4^. Endocrine therapy (ET) restrains ILC cell proliferation and is the mainstay of systemic treatment for lobular BC in the clinic^5,6^. Activating mutation of *ERBB2* has been associated with recurrent and metastatic ILC and poor prognosis^7–14^. Estrogen receptor (ER)-positive human BC cell lines with isogenically incorporated *ERBB2* mutation acquire resistance to growth inhibition by estrogen deprivation in vitro^15^. Accordingly, it is thought that *ERBB2* mutation mediates endocrine resistance in vitro^10,15^.

However, it is currently unknown whether or how much *ERBB2* mutation impacts on the otherwise exquisite clinical responsiveness of ILC cells to endocrine therapy, especially in early ILC. Assessment of the *ERBB2* mutation status is not a standard diagnostic procedure in the clinic^16^. Furthermore, assessment of tumor cell proliferation in early BC pre-treated with endocrine therapy requires that anti-hormonal therapy is initiated a short time before tumor resection. This is not usually done in the clinic. Short-term preoperative endocrine therapy (pET) does not improve outcome, but it offers an empirical readout for endocrine responsiveness by measuring the post-pET Ki67 cell proliferation index^17–19^. So far, pET has only been implemented in a limited number of prospective clinical trials, including the POETIC and ADAPT trial^17–22^. Whether or not *ERBB2* mutation impacts on tumor cell proliferation in early ILC treated with preoperative endocrine therapy has not yet been investigated in this context. Therefore, we extended our previous, retrospective exploratory analyses of BCs from the ADAPT trial (HR-positive/HER2-negative)^23^. Here we report on the relation between genetic alterations and tumor cell proliferation following pET, with a special reference to ILC and *ERBB2* mutation.

## Materials and methods

### Tumor specimens

Tumor tissues included *n* = 622 HR-positive/HER2-negative early BCs from patients enrolled in the West German Study Group (WSG) ADAPT trial (NCT01779206). This cohort corresponds to a subset of patients enrolled in the ADAPT trial (run-in phase)^21–23^. Study design details were reported previously^21,22,24^. Short-term preoperative endocrine therapy (pET; with tamoxifen [TAM] or aromatase inhibitors [AIs]) was administered for three weeks before tumor resection^21,22^. Oncotype DX recurrence score (RS) testing at baseline was performed at the laboratory of Genomic Health Inc (Redwood City, CA, USA). All tumors were subjected to prospective central pathology review (MHH, Hannover, Germany) for histological subtyping and prospective assessment of tumor cell proliferation (documented in 2012–2016). Histologic BC subtypes were determined in accordance with the criteria of the “World Health Organization (WHO) classification of tumours of the breast”, 4^th^ edition (2012)^25^. Histologic BC subtype calls were based on a consensus of three experts headed by H.K. and were aided by upfront E-cadherin immunohistochemistry (IHC), as described recently^26^. A comprehensive description of criteria for the diagnosis of ILC has been provided elsewhere^1,2^. From an initial set of *n* = 701 case, *n* = 79 cases were excluded from further molecular work-up (performed in 2018–2020) due to (i) missing Ki67 values, (ii) unavailable tissue blocks (returned to local centers upon clinical request), (iii) divergent histological subtypes at baseline or post-pET or controversial classification as either lobular or non-lobular BC, including cases classifiable as BC with mixed ductal/lobular features (*n* = 12), (iv) triple-negative hormone receptor status, (v) insufficient DNA amount and/or quality^23^. The total number of cases for the final statistical analysis was *n* = 622 (Table 1). The characteristics of the study population included in this retrospective molecular analysis were reported earlier^23^. This study was approved by the local ethic committee (MHH, Hannover, ID 2716–2015).

**Table 1:** Tumor collection and BC subtypes.

	All BC cases		Non-lobular BC		Lobular BC				
	*n*	(%)	*n*	(%)	*n*	(%)	*P*-value	Test	Notes
All cases	622	(100.0)	443	(100.0)	179	(100.0)			
Age at diagnosis									
Median (range) in yrs	54	(28-76)	54	(28-76)	57	(28-75)			
pT stage									
pT1	371	(59.6)	274	(61.8)	97	(54.2)	0.0712	FET	pT1 vs pT2+
pT2	223	(35.9)	154	(34.8)	69	(38.5)	**0.0247**	FET	pT1/2 vs pT3+
pT3	24	(3.9)	12	(2.7)	12	(6.7)			
pT4	2	(0.3)	1	(0.2)	1	(0.6)			
n.a.	2	(0.3)	2	(0.5)	0	(0.0)			
pN stage									
pN0	541	(87.0)	379	(85.5)	162	(90.5)	0.1439	FET	pN0 vs pN1+
pN1+	79	(12.7)	62	(14.0)	17	(9.5)			
n.a.	2	(0.3)	2	(0.5)	0	(0.0)			
Histological grade, baseline									
G1	46	(7.4)	33	(7.4)	13	(7.3)	**0.0017**	FET	G1/2 vs G3
G2	399	(64.1)	268	(60.5)	131	(73.2)			
G3	177	(28.5)	142	(32.1)	35	(19.5)			
n.a.	0	(0.0)	0	(0.0)	0	(0.0)			
ER status, baseline									
Negative	1	(0.2)	1	(0.2)	0	(0.0)	1.0000	FET	ER pos vs neg
Low expression^a^	0	(0.0)	0	(0.0)	0	(0.0)			
Positive	620	(99.6)	442	(99.8)	178	(99.4)			
n.a.	1	(0.2)	0	(0.0)	1	(0.6)			
ER status, post-pET									
Negative	0	(0.0)	0	(0.0)	0	(0.0)	-	FET	ER pos vs neg
Low expression^a^	1	(0.2)	0	(0.0)	1	(0.6)			
Positive	620	(99.6)	442	(99.8)	178	(99.4)			
n.a.	1	(0.2)	1	(0.2)	0	(0.0)			
PR status, baseline									
Negative	46	(7.4)	32	(7.2)	14	(7.8)	0.8657	FET	PR pos vs neg
Low expression^a^	27	(4.3)	22	(5.0)	5	(2.8)			
Positive	549	(88.3)	389	(87.8)	160	(89.4)			
n.a.	0	(0.0)	0	(0.0)	0	(0.0)			
PR status, post-pET									
Negative	137	(22.0)	99	(22.4)	38	(21.2)	0.8310	FET	PR pos vs neg
Low expression^a^	65	(10.5)	48	(10.8)	17	(9.5)			
Positive	420	(67.5)	296	(66.8)	124	(69.3)			
n.a.	0	(0.0)	0	(0.0)	0	(0.0)			
HER2 status, baseline (According to ASCO/CAP guidelines)									
Negative	615	(98.9)	437	(98.7)	178	(99.4)	0.3285	FET	HER2 pos vs neg
Positive	5	(0.8)	5	(1.1)	0	(0.0)			
n.a.	2	(0.3)	1	(0.2)	1	(0.6)			
HER2 status, post-pET (According to ASCO/CAP guidelines)									
Negative	613	(98.5)	435	(98.2)	178	(99.4)	0.4495	FET	HER2 pos vs neg
Positive	8	(1.3)	7	(1.6)	1	(0.6)			
n.a.	1	(0.2)	1	(0.2)	0	(0.0)			
Ki67, baseline									
0–9	72	(11.6)	42	(9.5)	30	(16.8)	**0.0127**	FET	Ki67 <10 vs ≥10
10–19	244	(39.2)	163	(36.8)	81	(45.2)			
20–34	222	(35.7)	171	(38.6)	51	(28.5)			
35–100	84	(13.5)	67	(15.1)	17	(9.5)			
n.a.	0	(0.0)	0	(0.0)	0	(0.0)			
Ki67, post-pET									
0–9	327	(52.6)	223	(50.4)	104	(58.1)	0.0919	FET	Ki67 <10 vs ≥10
10–19	186	(29.9)	134	(30.2)	52	(29.0)			
20–34	87	(14.0)	67	(15.1)	20	(11.2)			
35–100	22	(3.5)	19	(4.3)	3	(1.7)			
n.a.	0	(0.0)	0	(0.0)	0	(0.0)			
Ki67, dynamic categories (according to the POETIC trial^18^)									
Low–low	59	(9.5)	38	(8.6)	21	(11.7)	**0.0169**	CSTT	
High–low	268	(43.1)	185	(41.8)	83	(46.4)	**0.0235**	FET	high/high vs x/low
High–high	282	(45.3)	216	(48.7)	66	(36.9)			
Low–high^b^	13	(2.1)	4	(0.9)	9	(5.0)			
n.a.	0	(0.0)	0	(0.0)	0	(0.0)			
E-cadherin, baseline									
Negative	171	(27.5)	11	(2.5)	160	(89.4)	**<0.0001**	FET	E-cad pos vs neg
Positive	411	(66.1)	402	(90.7)	9	(5.0)			
n.a.	40	(6.4)	30	(6.8)	10	(5.6)			
E-cadherin, post-pET									
Negative	169	(27.2)	9	(2.0)	160	(89.4)	**<0.0001**	FET	E-cad pos vs neg
Positive	418	(67.2)	408	(92.1)	10	(5.6)			
n.a.	35	(5.6)	26	(5.9)	9	(5.0)			
Oncotype DX RS, baseline									
0–11	142	(22.8)	101	(22.8)	41	(22.9)	**0.0002**	FET	RS 0-25 vs 26-100
12–25	362	(58.2)	241	(54.4)	121	(67.6)			
26–100	101	(16.3)	87	(19.6)	14	(7.8)			
n.a.	17	(2.7)	14	(3.2)	3	(1.7)			
pET agent									
Tamoxifen	286	(46.0)	204	(46.0)	82	(45.8)	0.9294	FET	TAM vs AI
Aromatase inhibitors	334	(53.7)	237	(53.5)	97	(54.2)			
n.a.	2	(0.3)	2	(0.5)	0	(0.0)			

Unless otherwise stated, the values are given in the format *n* (%), with *n* corresponding to the number of patients. The Fisher’s exact test (FET) and Chi-Square test for trends (CSTT) were used for statistical analysis. Significant differences are highlighted in bold.

*n.a*. not available, *ER* estrogen receptor, *PR* progesterone receptor, *pET* preoperative endocrine therapy, *RS* recurrence score, *TAM* tamoxifen, AI aromatase inhibitors.

^a^Low expression (ER and PR status) was defined as 1–9% positive cells.

^b^Patients classified into low-high group were excluded for statistical analysis.

### Immunohistochemistry and assessment of tumor cell proliferation

Immunohistochemistry (IHC) for estrogen receptor (ER), progesterone receptor (PR) and HER2 was performed prospectively in the central pathology unit of the ADAPT trial as described previously^23^. BCs scored as HER2 2+ and 3+ were subjected to *HER2/ERBB2* fluorescence in situ hybridization in accordance with ASCO/CAP guidelines^16^. Tumor cell proliferation before endocrine therapy (baseline) and after endocrine therapy (post-pET) was determined prospectively by standardized central pathology assessment of the Ki67 cell proliferation marker^18,19,23^. Immunohistochemical staining of Ki67 was performed with the anti-Ki67 antibody clone 30-9 (Ventana, Tucson, AZ, USA)^23,27,28^. Ki67 scoring was supported by computer-assisted image analysis (iScan Coreo slide scanner and Virtuoso v5.3 software for digital quantification, Ventana, Tucson, AZ, USA)^29,30^. The Ki67 index was based on three independent evaluations (2x semiquantitative assessments by experienced pathologists, 1x digital quantification with Virtuoso software)^22,23^. The semiquantitative Ki67 index that was nearest to the digital Ki67 index was accepted as the consensus Ki67 index^22,23^. Representative immunohistochemical stainings of BCs in each Ki67 category (0–9%, 10–19%, 20–34%, and 35–100%) are shown in the data supplement (Supplementary Fig. 1A). The *MKI67* gene is one of the key determinants of the Oncotype DX recurrence scores^31^. Higher baseline Ki67 indices were well correlated with higher recurrence scores, which indirectly substantiated the validity of Ki67 assessment by IHC (Supplementary Fig. 1B)^17^. Sustained and suppressed tumor cell proliferation after therapy were defined as post-pET Ki67 ≥10% and <10%^23^. This cutoff represents a provisional cutoff that was implemented only for retrospective molecular analyses in the ADAPT translation research program^23^. This cutoff is consistent with similar analyses in the POETIC trial^18^. In the POETIC trial, post-pET Ki67 indices of ≥10% and <10% were termed high and low post-treatment proliferation, respectively^18^. E-cadherin protein expression was determined with the anti-E-cadherin antibody ECH-6 (Zytomed Systems, Berlin, Germany)^26^. E-cadherin IHC was scored as negative (complete loss) versus positive (any specific staining). All IHC stainings were performed on a Benchmark Ultra (Ventana, Tucson, AZ, USA) automated stainer.

### DNA extraction and mutational analysis

Extraction of DNA and analysis of genetic alterations were performed (in 2018-2020) as described previously^23^. In addition, mutational analysis of the *CDH1* gene was carried out by next generation sequencing (NGS) using a customized NGS panel, which covered the complete protein-coding sequence including 10 bp of the flanking intron sequence of the *CDH1* gene. Mean mapped reads per sample was 282,620 (range 33,486 to 7,149,110). Data processing and evaluation were performed as described previously^23^.

### Statistics

For statistical evaluation of the association between genetic alterations and pathologic parameters, we focused on candidate genes with a mutation frequency of ≥2.5%. The two-sided Fisher’s exact test and the Chi square test for trends were used for contingency analyses. The Mann–Whitney test was used to determine statistical significance of different median Ki67 indices in BC subsets. Results were considered as statistically significant if *P* ≤ 0.0500. Statistical analyses were performed with GraphPad Prism software Version 5.00 (GraphPad Software, San Diego, CA, USA).

## Results

### Baseline characteristics

We have recently profiled *n* = 622 HR-positive early BCs from the ADAPT trial for genetic alterations in selected candidate genes involved in endocrine resistance or responsiveness^23^. All patients were treated with short-term pET (TAM or AI) for three weeks before tumor resection^21,22^. Here, we report a subgroup analysis pertaining to the lobular BCs included in this cohort. According to central pathology review, lobular BC accounted for 179/622 (28.8%) cases. ILC was associated with larger pT stage, lower histological grade, lower Oncotype DX recurrence scores, lower baseline Ki67 (before pET), and loss of E-cadherin (Table 1). This is consistent with previous studies^32^.

### Genetic alterations in ILC

**Fig. 1:**
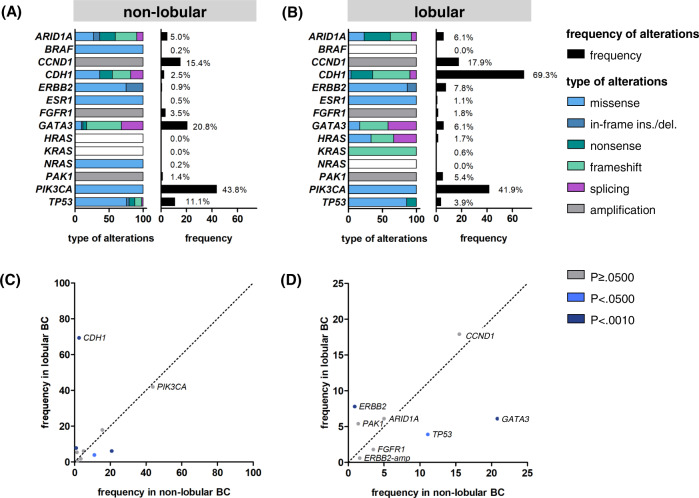
Mutation frequencies in lobular and non-lobular BC. Bar charts showing the frequencies of genetic alterations in candidate genes involved in endocrine tumor response in non-lobular (**A**) and lobular BC (**B**). A scatter plot illustrating the frequencies gene mutations in non-lobular (x-axis) versus lobular BC (y-axis) is shown in the lower panel (**C**). A magnified view of the inset is provided on the right side (**D**). The color of the dots reflect statistical significance (of frequencies in non-lobular BC versus lobular BC). Colored dots correspond to statistically significant differences with *P* values of <0.0500, and <0.0010, respectively. ins insertion, del deletion.

**Fig. 2:**
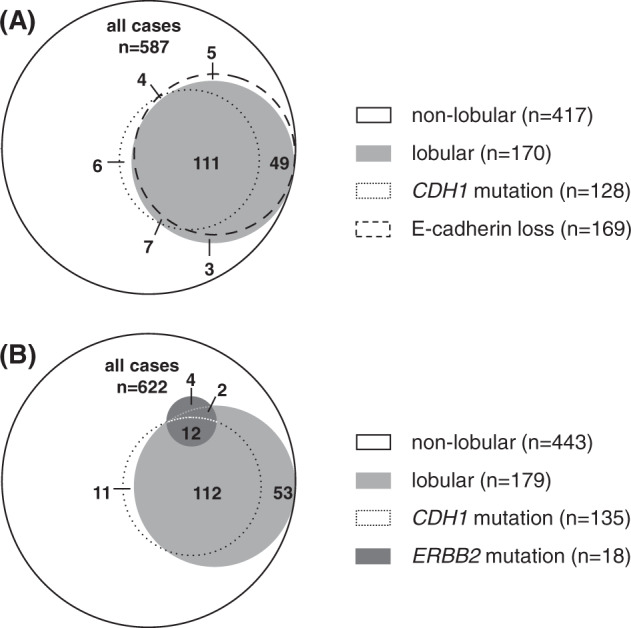
Lobular BC is associated with *CDH1* mutation, loss of E-cadherin, and *ERBB2* mutation. **A** Venn diagram showing the overlap between lobular BC, *CDH1* mutation, and loss of E-cadherin, as determined by IHC with the anti-E-cadherin antibody ECH-6. Please note that the E-cadherin IHC status was not available for 35/622 patients. **B** Venn diagram showing the overlap between lobular BC, *CDH1* mutation, and *ERBB2* mutation. Numbers within the Venn diagram indicate numbers of patients/cases.

Genes previously assessed in this cohort included *ARID1A*, *BRAF*, *ERBB2*, *ESR1*, *GATA3*, *HRAS*, *KRAS*, *NRAS*, *PIK3CA*, and *TP53* (mutational analysis by NGS), and *CCND1*, *FGFR1* and *PAK1* (copy number assessment by digital PCR)^23^. These genes were selected as candidate genes involved in endocrine tumor response based on the study of Razavi et al., which focused on metachronous BC recurrences after failure of (adjuvant) ET^10,23^. For the present subgroup analysis of ILCs we also performed mutational analyses of *CDH1*. As expected, ILCs were enriched in cases harboring mutations in *CDH1* (124/179, 69.3%, *P* < 0.0001) (Fig. 1A, B). In contrast, *CDH1* mutations were rare in non-lobular BC (11/443, 2.5%) (Fig. 1A, B). Furthermore ILCs were enriched in mutations in *ERBB2* (14/179, 7.8%, *P* < 0.0001), but showed fewer mutations in *TP53* (7/179, 3.9%, *P* = 0.0048) and *GATA3* (11/179, 6.1%, *P* < 0.0001) (Fig. 1C, D, Supplementary Table 1). Regarding mutation types, *CDH1* mutations were mostly nonsense and frameshift mutations (114/135, 84.4%) (Fig. 1B). *CDH1* mutations were nearly always accompanied by loss of E-cadherin (115/128, 89.8%) (Fig. 2A). *CDH1* missense mutations associated with preserved E-cadherin expression were rare (7/128, 5.5%) (Supplementary Fig. 2). Most *ERBB2* mutations were missense mutations (15/18, 83.3%) resulting in single amino acid substitutions in the tyrosine kinase domain, such as p.L755S (Fig. 1B and Supplementary Table 2). The remaining *ERBB2* mutations (3/18, 16.7%) were exon 20 in-frame insertions or duplications, such as p.Y772_A775dup, which are more common in non-small cell lung cancer (NSCLC) and induce a constitutively active ERBB2 protein conformation (Supplementary Table 2)^33^. One ILC (case 110290) harbored two different *ERBB*2 mutations (p.I767M and p.V777L) (Supplementary Table 2). In total, 12/18 (66.7%) *ERBB2*-mutated BCs harbored a concomitant *CDH1* mutation (*P* < 0.0001), confirming the strong association of these two alterations, as reported earlier (Fig. 2B)^8^. Overall, mutation frequencies were consistent with independent previous studies^3,34–36^. However, our BC cohort showed slightly more *CDH1* and *ERBB2* mutations in ILC, and more *GATA3* mutations in non-lobular BC than reported in previous studies (Supplementary Fig. 3 and Supplementary Table 3).

### Relation between post-pET Ki67 and *ERBB2* mutation in ILC

**Fig. 3:**
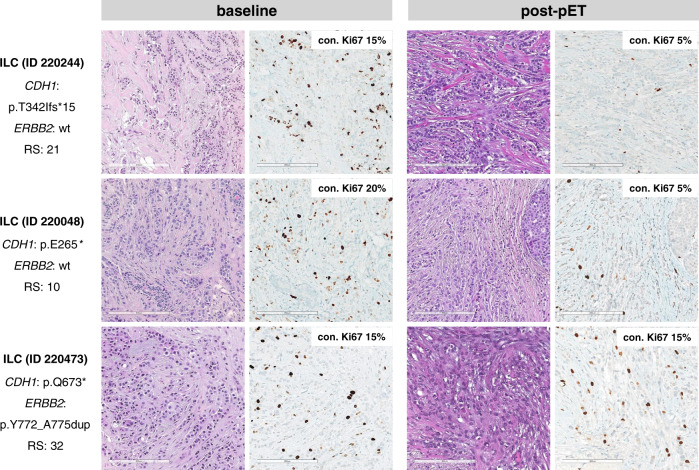
Representative immunohistochemical stainings for Ki67 at baseline and post-pET. Shown are three representative lobular BCs (IDs 220244, 220048, and 220473). Case IDs, *CDH1* and *ERBB2* mutations and Oncotype DX recurrence scores are indicated at the left margin. The left panels show HE- and Ki67-stained core needle biopsies (CNBs) before treatment (baseline) at x200 magnification (scale bar corresponds to 200 µm). The right panels show HE- and Ki67-stained resection specimens of the same tumors after per-operative endocrine therapy (post-pET). Insets in the upper right corners indicate the consensus Ki67 index (con. Ki67 [% positive tumor cells]) obtained by semiquantitative assessment by two experienced pathologist and digital quantification using computer-assisted image analysis (Virtuoso v5.3 software), as described in the materials and methods section. con consensus, RS recurrence score, wt wild-type.

An advantage of the BC collection described herein is that patients were treated with short-term pET before surgery^21^. Furthermore, post-pET tumor cell proliferation was prospectively determined by standardized central assessment of the Ki67 index in the framework of a controlled clinical trial (ADAPT)^21^. Moreover, central Ki67 scoring was supported by computer-assisted image analysis^23^. No such comprehensive information on post-pET proliferation is available for specimens in public BC mutation databases, such as METABRIC or TCGA^4^. Representative immunohistochemical stainings for Ki67 at baseline and post-pET are shown in Fig. 3 (Fig. 3). Our previous analyses in this BC cohort (*n* = 622) revealed that sustained tumor cell proliferation after preoperative endocrine therapy (defined as post-pET Ki67 ≥10%) was associated with *TP53* mutation but not with *ERBB2* mutation^23^. In fact, by considering all BCs irrespective of subtypes, *ERBB2* mutation was not associated with any prognostic parameter or enhanced proliferation (Supplementary Table 4). This was surprising, given that mutant *ERBB2* mediates estrogen-independent proliferation in ER-positive BC cell lines in vitro^15^. *ERBB2* mutation was also not associated with lower ER or PR expression or higher histological grade at baseline (before pET) (Supplementary Figs. 4 and 5, and Supplementary Table 4).

**Fig. 4:**
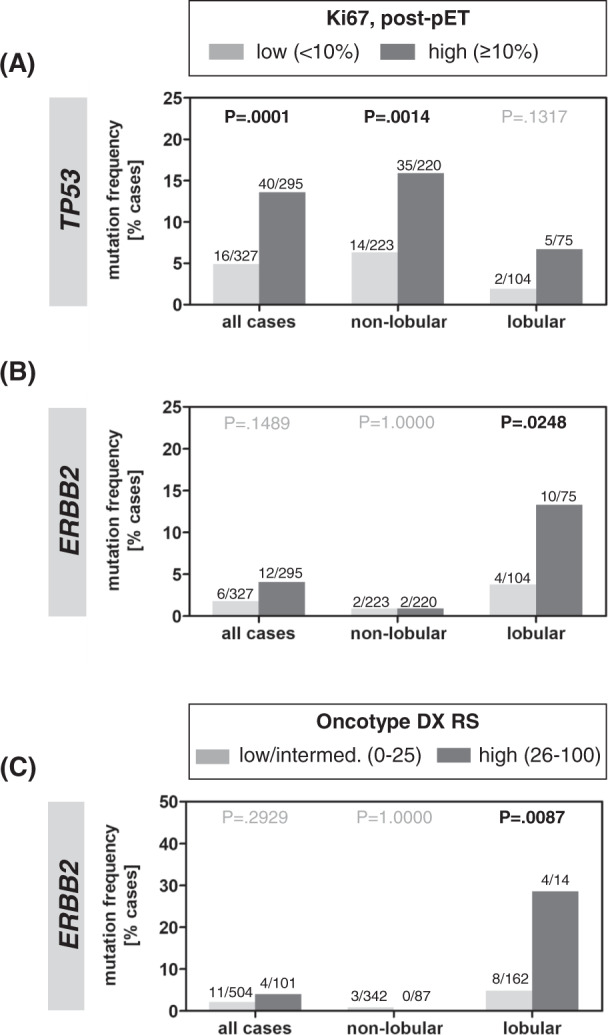
Relation between *TP53* or *ERBB2* mutation and post-pET Ki67 in lobular and non-lobular BC. **A** Bar chart showing the *TP53* mutation frequency among cases with post-pET Ki67 < 10% (gray) or ≥10% (dark gray). **B** Bar chart showing the *ERBB2* mutation frequency among cases with post-pET Ki67 < 10% (gray) or ≥10% (dark gray). **C** Bar chart showing the *ERBB2* mutation frequency among cases with low/intermediate Oncotype DX recurrence score (RS 0–11 plus RS 12–25, light gray), or high recurrence score (RS 26–100, dark gray). Statistical significance was determined with Fisher’s Exact Test. RS recurrence score, pET preoperative endocrine therapy.

As *ERBB2* mutation occurred mostly in ILC, and because lobular BCs are mostly slow growing tumors, we hypothesized that the impact of *ERBB2* mutation on post-pET proliferation might not be apparent, if ILC and non-lobular BCs are lumped together for statistical analysis. Accordingly, we conducted exploratory subgroup analyses to specify genetic alterations associated with post-pET Ki67 in lobular and non-lobular BC respectively (Supplementary Table 5). Strikingly, sustained tumor cell proliferation after preoperative endocrine therapy (post-pET Ki67 ≥10%) was associated with *TP53* mutation in non-lobular BC (Fig. 4A) but with *ERBB2* mutation in ILC (Fig. 4B). In detail, *ERBB2* mutations were 3.5-fold more common in ILCs with sustained post-pET proliferation compared to ILCs with suppressed post-pET proliferation (10/75, 13.3% *versus* 4/104, 3.8%, *P* = 0.0248) (Fig. 4B). Conversely, failure of pET to achieve optimal suppression of tumor cell proliferation (post-pET Ki67 < 10%) was 1.8-fold more common in *ERBB2*-mutated ILC compared to ILC harboring wild-type *ERBB2* (10/14, 71% *versus* 65/165, 39%, *P* = 0.0248). This association was also statistically significant in the subset of ILC patients that were treated with AI for pET (Supplementary Table 5). Furthermore, *ERBB2* mutations in ILCs were associated with the “high-high” dynamic Ki67 category (baseline and post-pET Ki67 both ≥10%), as defined recently in the POETIC trial (*P* = 0.0263) (Supplementary Fig. 6 and Supplementary Table 6)^18^. ILCs harboring *ERBB2* mutations also showed a borderline significant trend towards a higher median post-pET Ki67 index (*P* = 0.0513) (Supplementary Fig. 7). Particularly high baseline and post-pET Ki67 indices (both 30%) were observed in one ILC that harbored two different *ERBB2* mutations (case 110290) (Supplementary Table 2). Moreover, *ERBB2* mutation was associated with high risk Oncotype DX recurrence scores (*P* = 0.0087), but only in the subgroup of lobular BCs (Fig. 4C).

### Concordant detection of *ERBB2* mutation in matched baseline and post-pET specimens

*ERBB2* mutations may arise de novo during endocrine therapy^10^. In our BC cohort, *ERBB2* mutations were concordantly detected in resections specimens (post-pET) and matched core needle biopsies (baseline, before pET) in 14/18 (77.8%) cases tested (Supplementary Table 2). This indicates that most *ERBB2* mutations were already present at baseline, in treatment-naïve BCs.

## Discussion

Lobular BC is a distinct tumor entity characterized by a special histomorphology, distinct genetic alterations, including *CDH1* mutation, and comparatively slow, estrogen-dependent growth^1,3,4^. Activating mutation of *ERBB2* has been associated with recurrent and metastatic ILC and poor prognosis^7–12^. Based on in vitro models, it is thought that mutant *ERBB2* mediates hormone-independent cell proliferation and thus resistance to endocrine therapy^15^. This appears to explain the overrepresentation of *ERBB2*-mutated ILC among patients with tumor recurrences after adjuvant endocrine therapy^7,10^. However, up to now, there was only limited or no evidence that *ERBB2* mutation actually does make a change for tumor cell proliferation in patients receiving endocrine therapy, especially in early lobular BC. The limited availability of tumor specimens from patients that have been treated with short-term pET before surgery for early BC may be one of the reasons for the lack of studies in this direction^17–19^.

In the present study we have extended our previous molecular analyses of BC specimens from the ADAPT (HR-positive/HER2-negative) trial, in which all patients were treated with pET before surgery^21–23^. ILCs included in this cohort were strongly enriched in *CDH1* mutations and *ERBB2* mutations. This is in line with previous studies^3,8,34–36^. Considering all BCs irrespective of subtypes, *ERBB2* mutation did not show any significant association with prognostic parameters or tumor cell proliferation. In the subgroup of ILCs, however, *ERBB2* mutations were enriched 3.5-fold in cases with sustained post-pET proliferation compared to cases with suppressed post-pET proliferation (13.3% versus 3.8%, *P* = 0.0248). Moreover, *ERBB2* mutation was associated with high risk Oncotype DX recurrences scores, but only in the subgroup of lobular BC.

Limitations of the present study include: (i) the retrospective approach of molecular analyses, (ii) the exploratory subgroup analysis for BCs with lobular histology, (iii) exclusion of a small minority of cases with controversial subtype calls (including cases classifiably as BC with mixed ductal/lobular features) and (iv) the comparatively small absolute number of *ERBB2*-mutated cases. However, compared with the situation in early HR-positive/HER2-positive BCs, there is a lack of prospective clinical trials designed specifically for early HR-positive and/or *ERBB2*-mutant ILC. Accordingly, retrospective exploratory subgroup analyses may represent a necessary intermediate step on the way towards new clinical trials for this distinct tumor type.

In fact, the findings reported here are important for two reasons. First, this study further supports that mutant *ERBB2* influences endocrine responsiveness in lobular BC. For the first time, this has now also been demonstrated by means of clinical tumor specimens obtained after pET. Even so, a proportion of *ERBB2-*mutant ILCs still showed optimal suppression of tumor cell proliferation by pET. Accordingly, it remains an open question whether *ERBB2* mutation is a suitable prognostic factor that is independent from other parameters, such as the Onctotype DX recurrence score. Further analyses in larger cohorts treated with adjuvant endocrine therapy alone, or with adjuvant endocrine therapy plus adjuvant chemotherapy would be necessary to clarify this issue. The current retrospective, exploratory subgroup analysis of a proportion of patients enrolled in the ADAPT trial (run-in phase) was not sufficiently powered to address this problem. Second, this subgroup analysis exemplifies that biologically relevant associations may be masked and can remain unidentified, if HR-positive ILCs and non-lobular BCs are lumped together for statistical analyses in BC research. Accordingly, lobular BC deserves greater attention in clinical trials and translational research.

## Supplementary information

Supplemental Material

## Data Availability

The datasets used and analyzed during the current study are available from the corresponding author on reasonable request.
