# Supplementary material for: ERBB2 mutation is associated with sustained tumor cell proliferation after short-term preoperative endocrine therapy in early lobular breast cancer
Source: Mod Pathol. 2022 Jul 16;35(12):1804–11. doi: 10.1038/s41379-022-01130-7 (PMC9708567; doi:10.1038/s41379-022-01130-7)
Supplement: Supplementary file 1 — Supplemental Material [file 41379_2022_1130_MOESM1_ESM.pdf]

Data Supplement

ERBB2 mutation is associated with sustained tumor cell proliferation after short-term preoperative endocrine therapy in early lobular breast cancer

Grote *et al.* 2022

Table of contents

Supplemental Figure 1. Representative immunohistochemical stainings for Ki67 categories. 3

Supplemental Figure 2. Agreement between E-cadherin IHC and *CDH1* mutation. 4

Supplemental Figure 3. Mutational characteristics are consistent with previous studies. 5

Supplemental Figure 4. Expression of ER is similar in lobular BCs with mutated or wild-type *ERBB2*. 6

Supplemental Figure 5. Expression of PR is similar in lobular BCs with mutated or wild-type *ERBB2*. 7

Supplemental Figure 6. Relation between dynamic Ki67 categories and *TP53* or *ERBB2* mutation in lobular and non-lobular BC. 8

Supplemental Figure 7. Distribution of Ki67 indices in BCs with *ERBB2* wild-type or *ERBB2* mutant status. 9

Supplemental Table 1. Genetic alterations in lobular and non-lobular BC 10

Supplemental Table 2. Details of all *ERBB2*-mutated BCs. 11

Supplemental Table 3. Mutational characteristics are consistent with previous studies 12

Supplemental Table 4. Clinicopathological parameters associated with *ERBB2*-mutated BC. 14

Supplemental Table 5. Genetic alterations and their distribution according to post-pET Ki67. 16

Supplemental Table 6. Genetic alterations and their distribution according to dynamic Ki67 categories. 18

Supplemental Figure 1. Representative immunohistochemical stainings for Ki67 categories.

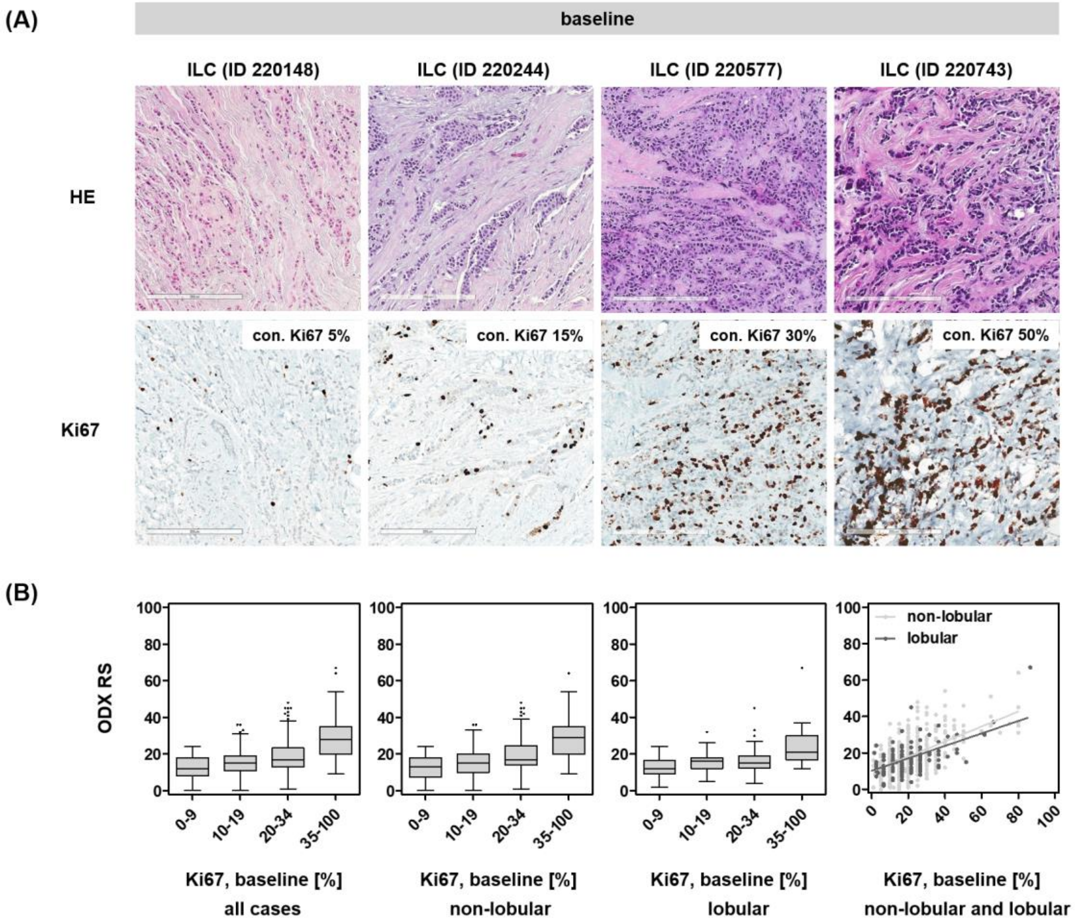

(A) Representative HE- and Ki67-stained sections of lobular BCs in each Ki67 category (0-9%, 10-19%, 20-34%, and 35-100%) at x200 magnification. (B) Box plots showing increasing median Oncotype DX RSs according to increasing baseline Ki67 categories. Boxes indicate interquartile ranges, whiskers indicate 1.5-fold interquartile distances. From left to right, plots correspond to all BC, non-lobular BC and lobular BC. Correlation analyses for baseline Ki67 and RS are shown in the far right panel. Spearman correlation coefficients were r=0.47 (95% CI: 0.39-0.54, P<.0001) for non-lobular BC and r=0.34 (95% CI: 0.19-0.46, P<.0001) for lobular BC. <u>Abbreviations:</u> HE, hematoxylin/eosin; con, consensus; ODX RS, Oncotype DX recurrence score; CI, confidence interval.

Supplemental Figure 2. Agreement between E-cadherin IHC and *CDH1* mutation.

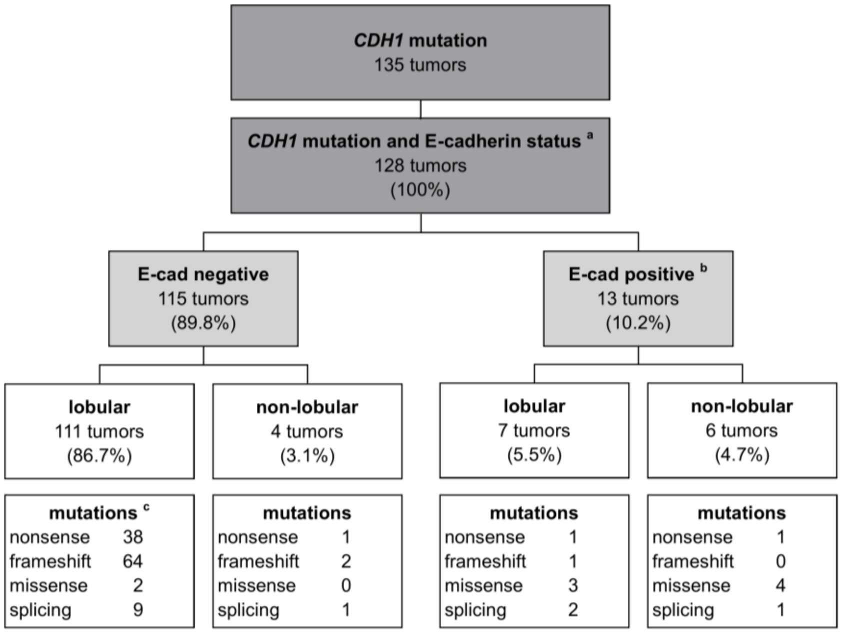

Based on E-cadherin and histological type, the tree diagram shows the distribution of tumors with *CDH1* mutation.

[a] For seven *CDH1*-mutated BCs the E-cadherin status was not available.

[b] E-cadherin positive and *CDH1*-mutated BCs showed aberrant, fragmented E-cadherin immunoreactivity.

[c] Two E-cadherin negative lobular BCs harbored each two *CDH1* mutations (missense and nonsense, respectively frameshift).

<u>Abbreviations:</u> E-cad, E-cadherin.

Supplemental Figure 3. Mutational characteristics are consistent with previous studies.

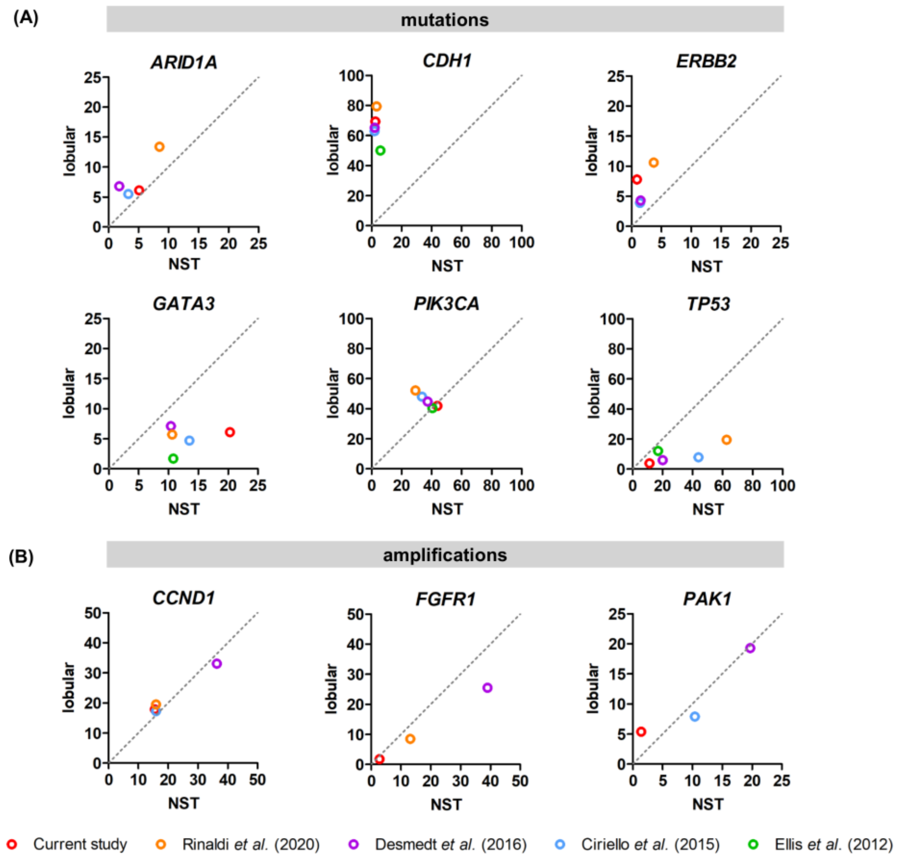

Frequencies of genetic alteration for BCs of NST and lobular BC in the current study (red) and in previous studies. For comparison data were retrieved from the studies of Desmedt *et al.* (2016, purple) and Ellis *et al.* (2012, green) describing HR-positive/HER2-negative BC collections. Further data were retrieved from the studies of Rinaldi *et al.* (2020, gold) and Ciriello *et al.* (2015, blue) describing unselected BC collections. For some genetic alterations, data were not available in all four studies. Abbreviations: NST, no special type

Supplemental Figure 4. Expression of ER is similar in lobular BCs with mutated or wild-type *ERBB2*.

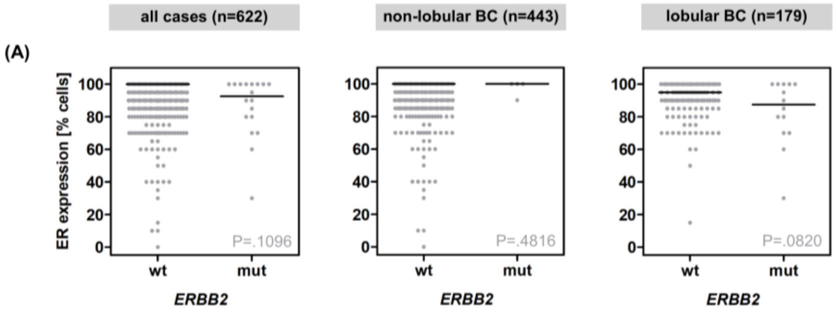

	all cases wt	all cases mut	non-lobular BC wt	non-lobular BC mut	lobular BC wt	lobular BC mut
	n=603	n=18	n=439	n=4	n=164	n=14
min	0.0	30.0	0.0	90.0	15.4	30.0
25%	90.0	77.5	90.0	92.5	90.0	70.0
median	100.0	92.5	100.0	92.5	95.0	87.5
75%	100.0	100.0	100.0	100.0	100.0	100.0
max	100.0	100.0	100.0	100.0	100.0	100.0

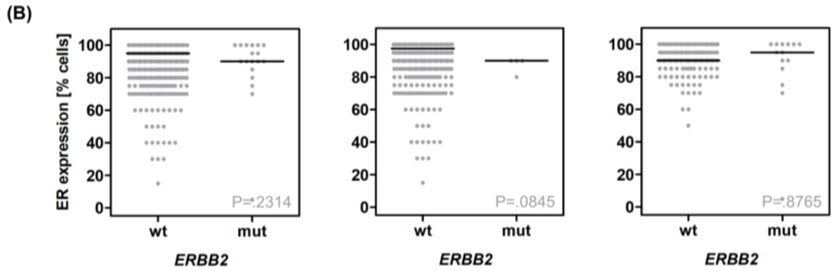

	all cases wt	all cases mut	non-lobular BC wt	non-lobular BC mut	lobular BC wt	lobular BC mut
	n=603	n=18	n=438	n=4	n=165	n=14
min	15.0	5.0	15.0	80.0	50.0	5.0
25%	90.0	83.8	90.0	82.5	90.0	82.5
median	95.0	90.0	97.5	90.0	90.0	95.0
75%	100.0	100.0	100.0	100.0	100.0	100.0
max	100.0	100.0	100.0	100.0	100.0	100.0

(A) Scatter dot plots showing the baseline ER expression (as determined by IHC) of BCs with either *ERBB2* wild-type status or *ERBB2* mutation among cases. Note that ER expression was not available for 1/179 patients with lobular BC. (B) Scatter dot plots showing the post-pET ER expression of BCs with either *ERBB2* wild-type status or *ERBB2* mutation among cases. Note that ER expression was not available for 1/443 patients with non-lobular BC. From left to right, plots correspond to all BC, non-lobular BC and lobular BC, respectively. Statistical significance was determined with the Mann Whitney test.

Abbreviations: wt, cases with wild-type *ERBB2;* mut, cases with *ERBB2* mutations; IHC, immunohistochemistry; pET, preoperative endocrine therapy

Supplemental Figure 5. Expression of PR is similar in lobular BCs with mutated or wild-type *ERBB2*.

(A)

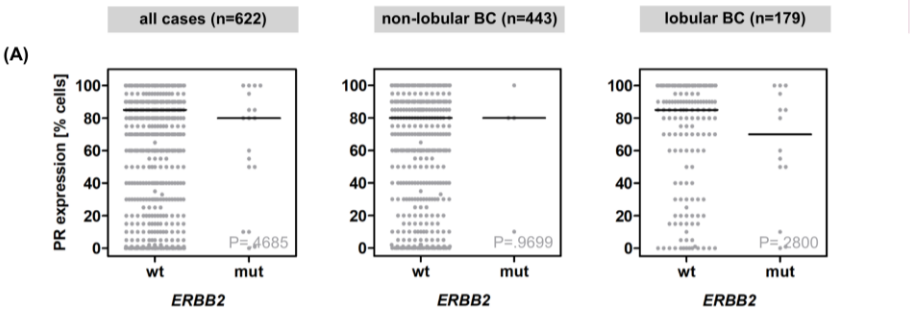

all cases (n=622)

	wt	mut
	n=604	n=18
min	0.0	0.0
25%	40.0	40.0
median	85.0	80.0
75%	100.0	96.3
max	100.0	100.0

non-lobular BC (n=443)

	wt	mut
	n=439	n=4
min	0.0	10.0
25%	40.0	27.5
median	80.0	80.0
75%	100.0	95.0
max	100.0	100.0

lobular BC (n=179)

	wt	mut
	n=165	n=14
min	0.0	0.0
25%	45.0	40.0
median	85.0	70.0
75%	100.0	96.3
max	100.0	100.0

(B)

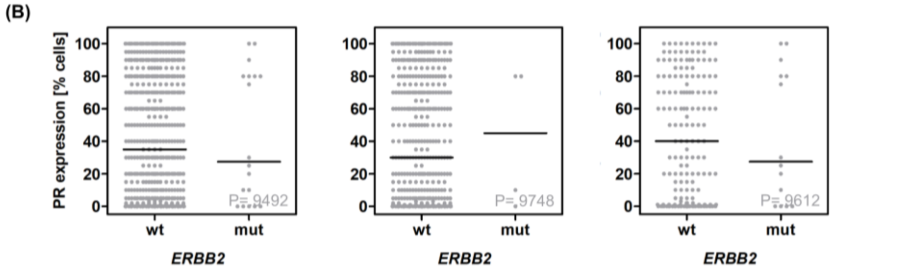

all cases (n=622)

	wt	mut
	n=603	n=18
min	0.0	0.0
25%	1.0	0.0
median	35.0	27.5
75%	80.0	80.0
max	100.0	100.0

non-lobular BC (n=443)

	wt	mut
	n=438	n=4
min	0.0	0.0
25%	1.0	2.5
median	30.0	45.0
75%	80.0	80.0
max	100.0	80.0

lobular BC (n=179)

	wt	mut
	n=165	n=14
min	0.0	0.0
25%	1.0	0.0
median	40.0	27.5
75%	80.0	82.5
max	100.0	100.0

(A) Scatter dot plots showing the baseline PR expression (as determined by IHC) of BCs with either *ERBB2* wild-type status or *ERBB2* mutation among cases.

(B) Scatter dot plots showing the post-pET PR expression of BCs with either *ERBB2* wild-type status or *ERBB2* mutation among cases. Note that PR expression was not available for 1/443 patients with non-lobular BC. From left to right plots correspond to all BC, non-lobular BC and lobular BC, respectively. Statistical significance was determined with Mann Whitney test.

Abbreviations: wt, cases with wild-type *ERBB2;* mut, cases with *ERBB2* mutations; IHC, immunohistochemistry; pET, preoperative endocrine therapy

Supplemental Figure 6. Relation between dynamic Ki67 categories and *TP53* or *ERBB2* mutation in lobular and non-lobular BC.

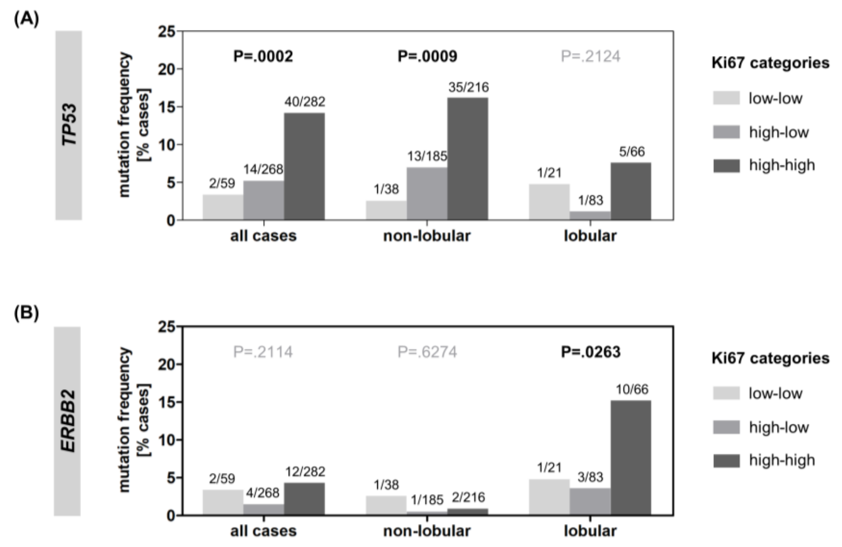

According to Ki67 indices BCs were classified four categories: (i) low-low (baseline and post-pET Ki67 <10%, light grey), (ii) high-low (baseline Ki67 ≥10%, and post-pET Ki67 <10%, grey), (iii) high-high (baseline and post-pET Ki67 both ≥10%, dark grey), and low-high (baseline Ki67 <10%, and post-pET Ki67 ≥10%, data not shown because of the low number cases in this category, as indicated in Table 1). This cutoff and this categorization is consistent with similar analyses in the POETIC trial (Smith *et al.* 2020).

(A) Bar chart showing the *TP53* mutation frequency according to dynamic Ki67 categories. (B) Bar chart showing the *ERBB2* mutation frequency according to dynamic Ki67 categories. From left to right, bar charts correspond to all BC, non-lobular BC and lobular BC, respectively. Statistical significance was determined with Chi-Square Test for Trends.

Supplemental Figure 7. Distribution of Ki67 indices in BCs with *ERBB2* wild-type or *ERBB2* mutant status.

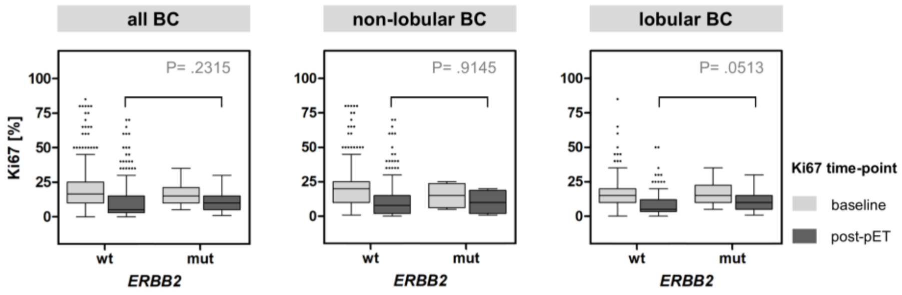

Box plots showing median Ki67 indices according to time point (baseline *versus* post-pET) and according to *ERBB2* mutation status. Boxes indicate the interquartile ranges, whiskers indicate 1.5-fold interquartile distances. From left to right, box plots correspond to all BC, non-lobular BC and lobular BC, respectively. Statistical significance was determined with the Mann Whitney test.

Abbreviations: wt, cases with wild-type *ERBB2;* mut, cases with *ERBB2* mutations; pET, preoperative endocrine therapy

Supplemental Table 1. Genetic alterations in lobular and non-lobular BC

Gene mutations were analyzed in the whole cohort. Gene amplifications (*CCND1, FGFR1*, and *PAK1*) were determined in a subcohort of 199 cases with cellularity >50%. The Fisher's Exact Test was used for statistical analysis. Significant values are highlighted in bold.

[a] Non-lobular BCs included NST (n=434), mucinous (n=5), cribriform (n=1) and tubular (n=3) BC.

Abbreviations: NST, no special type; wt, wild-type; mut, mutations

	non-lobular BC[a] (n=443)				NST (n=434)				lobular BC (n=179)				lobular vs non-lobular	lobular vs NST
	wt	(%)	mut	(%)	wt	(%)	mut	(%)	wt	(%)	mut	(%)		
mutations														
ARID1A	421	(95.0)	22	(5.0)	412	(94.9)	22	(5.1)	168	(93.9)	11	(6.1)	.5565	.5615
CDH1	432	(97.5)	11	(2.5)	423	(97.5)	11	(2.5)	55	(30.7)	124	(69.3)	**<.0001**	**<.0001**
ERBB2	439	(99.1)	4	(0.9)	430	(99.1)	4	(0.9)	165	(92.2)	14	(7.8)	**<.0001**	**<.0001**
GATA3	351	(79.2)	92	(20.8)	346	(79.7)	88	(20.3)	168	(93.9)	11	(6.1)	**<.0001**	**<.0001**
PIK3CA	249	(56.2)	194	(43.8)	244	(56.2)	190	(43.8)	104	(58.1)	75	(41.9)	.7208	.7200
TP53	394	(88.9)	49	(11.1)	385	(88.7)	49	(11.3)	172	(96.1)	7	(3.9)	**.0048**	**.0031**
amplifications														
CCND1	121	(84.6)	22	(15.4)	120	(84.5)	22	(15.5)	46	(82.1)	10	(17.9)	.6718	.6731
ERBB2	436	(98.4)	7	(1.6)	427	(98.4)	7	(1.6)	178	(99.4)	1	(0.6)	.4496	.4481
FGFR1	138	(96.5)	5	(3.5)	138	(97.2)	4	(2.8)	55	(98.2)	1	(1.8)	1.0000	1.0000
PAK1	141	(98.6)	2	(1.4)	140	(98.6)	2	(1.4)	53	(94.6)	3	(5.4)	.1366	.1384

Supplemental Table 2. Details of all *ERBB2*-mutated BCs.

[a] Histological type baseline and post-pET.

[b] Not available due to insufficient tissue amount.

[c] Mutation status detected by pyrosequencing.

<u>Abbreviations:</u> pET, preoperative endocrine therapy; ER, estrogen receptor; PR, progesterone receptor; IHC, immunohistochemistry; FISH, fluorescence in situ hybridization; E-cad, E-cadherin; RS, Oncotype DX Recurrence Score; AF, allele frequency; TAM, tamoxifen; AI, aromatase inhibitors (anastrozole, letrozole and exemestane); n.a., not available; wt, *ERBB2* wild-type

			**baseline**								**post-pET**							**baseline**		**post-pET**		
**case ID**	**histological type**	**pET agent**	ER	PR	HER2 IHC	HER2 FISH	E-cad	Ki67	grade	RS	ER	PR	HER2 IHC	HER2 FISH	E-cad	Ki67	grade	*ERBB2* mutation	AF	*ERBB2* mutation	AF	Other alterations
invasive breast cancer of no specific type																						
110098	NST	TAM	100	100	2	0	1	20	2	n.a.	90	80	1	n.a.	1	20	2	p.V777L	36.1%	p.V777L	30.1%	-
110153	NST	TAM	100	80	2	0	1	25	2	15	80	80	2	0	1	15	2	n.a.[b]		p.S760F	2.4%	-
110244	NST	AI	100	10	0	n.a.	1	5	2	24	90	0	0	n.a.	1	5	1	p.G778_P780dup	39.9%	p.G778_P780dup	21.0%	-
220231	NST	AI	90	80	1	n.a.	1	10	1	15	90	10	0	n.a.	1	1	1	p.L755S	~15%[c]	p.L755S	30.0%	*PIK3CA*
invasive lobular breast cancers																						
110086	lobular	TAM	100	80	0	n.a.	0	10	2	n.a.	100	80	0	n.a.	0	10	2	p.L755S	~10%[c]	p.L755S	20.0%	*CDH1*
110132	lobular	AI	100	50	1	n.a.	0	15	2	9	100	80	1	n.a.	0	5	2	p.L755S	~15%[c]	p.L755S	40.0%	*CDH1*
110281	lobular	AI	70	85	1	n.a.	0	10	2	26	90	0	1	n.a.	0	5	2	p.V777L	14.0%	p.V777L	7.6%	*CDH1*
110290	lobular	TAM	100	100	2	0	0	30	3	n.a.	90	100	2	1	0	30	3	p.I767M<br>p.V777L	43.0%<br>45.4%	p.I767M<br>p.V777L	49.4%<br>47.1%	-
220042	lobular	AI	100	50	0	n.a.	0	5	2	11	100	10	1	n.a.	0	1	1	wt		p.P780S	3.1%	*ARID1A*
220225	lobular	AI	90	85	0	n.a.	0	15	2	15	100	100	0	n.a.	0	15	2	n.a.[b]		p.L869R	35.5%	*CDH1*
220473	lobular	AI	60	10	2	0	0	15	2	32	70	0	2	0	0	15	2	p.Y772_A775dup	34.9%	p.Y772_A775dup	26.4%	*CDH1*
220552	lobular	AI	30	0	2	0	0	20	2	45	5	0	1	n.a.	0	15	2	p.D769Y	58.0%	p.D769Y	26.8%	*CDH1*
220564	lobular	TAM	85	55	1	n.a.	0	15	2	21	85	30	1	n.a.	0	15	3	n.a.[b]		p.I767M	2.6%	*CDH1, GATA3* and *PIK3CA*
220602	lobular	TAM	70	100	0	n.a.	n.a.	10	2	19	75	75	1	n.a.	0	15	2	p.L755S	~10%[c]	p.L755S	10.0%	*CDH1*
220955	lobular	AI	80	1	1	n.a.	0	10	2	22	100	0	1	n.a.	0	5	2	p.V777L	23.7%	p.V777L	23.3%	*CCND1, CDH1* and *PIK3CA*
221046	lobular	TAM	80	95	2	n.a.	0	30	3	16	95	90	1	n.a.	0	10	2	p.L755S	~30%[c]	p.L755S	30.0%	*CDH1*
221073	lobular	AI	95	60	1	n.a.	0	35	3	26	95	25	1	n.a.	0	10	2	p.V842I	1.0%	p.V842I	2.8%	*CCND1, CDH1* and *KRAS*
221085	lobular	AI	100	100	0	n.a.	n.a.	10	2	6	100	20	0	n.a.	0	10	1	p.A775_G776insVA	11.5%	p.A775_G776insVA	24.1%	*CDH1* and *TP53*

Supplemental Table 3. Mutational characteristics are consistent with previous studies

The values are given in the format n (%), with n corresponding to the number of patients. For comparison, data were retrieved from the studies of Desmedt *et al.* (2016) and Ellis *et al.* (2012) describing HR-positive/HER2-negative BC collections. Further data were retrieved from Rinaldi *et al.* (2020) and Ciriello *et al.* (2015) describing unselected BC collections. For some genetic alterations, only frequencies were available. In such instances, the number of patients was stated as "not specified". For other genetic alterations, data were not available in all four studies cited above.

Abbreviations: NST, no special type; mut, mutations in the gene under consideration; wt, wild-type for the gene under consideration; n.s.; not specified; n.a.; no data available for this alteration

			NST						lobular BC					
			all cases		wt		mut		all cases		wt		mut	
		year	n	(%)	n	(%)	n	(%)	n	(%)	n	(%)	n	(%)
Mutations														
ARID1A	Current study	2022	434	(100.0)	412	(94.9)	22	(5.1)	179	(100.0)	168	(93.9)	11	(6.1)
	Rinaldi *et al.*	2020	4896	(100.0)	4478	(91.5)	418	(8.5)	611	(100.0)	529	(86.6)	82	(13.4)
	Desmedt *et al.*	2016	338	(100.0)	n.s.	(98.2)	n.s.	(1.8)	371	(100.0)	n.s.	(93.2)	n.s.	(6.8)
	Ciriello *et al.*	2015	490	(100.0)	474	(96.7)	16	(3.3)	127	(100.0)	120	(94.5)	7	(5.5)
	Ellis *et al.*	2012	259	(100.0)	n.a.		n.a.		58	(100.0)	n.a.		n.a.	
CDH1	Current study	2022	434	(100.0)	423	(97.5)	11	(2.5)	179	(100.0)	55	(30.7)	124	(69.3)
	Rinaldi *et al.*	2020	4896	(100.0)	n.s.	(96.7)	n.s.	(3.3)	611	(100.0)	n.s.	(20.6)	n.s.	(79.4)
	Desmedt *et al.*	2016	338	(100.0)	n.s.	(97.9)	n.s.	(2.1)	371	(100.0)	n.s.	(34.8)	n.s.	(65.2)
	Ciriello *et al.*	2015	490	(100.0)	480	(98.0)	10	(2.0)	127	(100.0)	47	(37.0)	80	(63.0)
	Ellis *et al.*	2012	169	(100.0)	159	(94.1)	10	(5.9)	40	(100.0)	20	(50.0)	20	(50.0)
ERBB2	Current study	2022	434	(100.0)	430	(99.1)	4	(0.9)	179	(100.0)	165	(92.2)	14	(7.8)
	Rinaldi *et al.*	2020	4896	(100.0)	n.s.	(96.3)	n.s.	(3.7)	611	(100.0)	n.s.	(89.4)	n.s.	(10.6)
	Desmedt *et al.*	2016	338	(100.0)	n.s.	(98.5)	n.s.	(1.5)	371	(100.0)	n.s.	(95.7)	n.s.	(4.3)
	Ciriello *et al.*	2015	490	(100.0)	483	(98.6)	7	(1.4)	127	(100.0)	122	(96.1)	5	(3.9)
	Ellis *et al.*	2012	259	(100.0)	n.a.		n.a.		58	(100.0)	n.a.		n.a.	
GATA3	Current study	2022	434	(100.0)	346	(79.7)	88	(20.3)	179	(100.0)	168	(93.9)	11	(6.1)
	Rinaldi *et al.*	2020	4896	(100.0)	4378	(89.4)	518	(10.6)	611	(100.0)	576	(94.3)	35	(5.7)
	Desmedt *et al.*	2016	338	(100.0)	n.s.	(89.6)	n.s.	(10.4)	371	(100.0)	n.s.	(93.0)	n.s	(7.0)
	Ciriello *et al.*	2015	490	(100.0)	424	(86.5)	66	(13.5)	127	(100.0)	121	(95.3)	6	(4.7)
	Ellis *et al.*	2012	259	(100.0)	231	(89.2)	28	(10.8)	58	(100.0)	57	(98.3)	1	(1.7)
PIK3CA	Current study	2022	434	(100.0)	244	(56.2)	190	(43.8)	179	(100.0)	104	(58.1)	75	(41.9)
	Rinaldi *et al.*	2020	4896	(100.0)	3468	(70.8)	1428	(29.2)	611	(100.0)	292	(47.8)	319	(52.2)
	Desmedt *et al.*	2016	338	(100.0)	n.s.	(62.7)	n.s.	(37.5)	371	(100.0)	n.s.	(55.3)	n.s.	(44.7)
	Ciriello *et al.*	2015	490	(100.0)	326	(66.5)	164	(33.5)	127	(100.0)	66	(52.0)	61	(48.0)
	Ellis *et al.*	2012	257	(100.0)	153	(59.5)	104	(40.5)	57	(100.0)	34	(59.6)	23	(40.4)
TP53	Current study	2022	434	(100.0)	385	(88.7)	49	(11.3)	179	(100.0)	172	(96.1)	7	(3.9)
	Rinaldi *et al.*	2020	4896	(100.0)	1827	(37.3)	3069	(62.7)	611	(100.0)	492	(80.5)	119	(19.5)
	Desmedt *et al.*	2016	338	(100.0)	n.s.	(79.9)	n.s.	(20.1)	371	(100.0)	n.s.	(94.1)	n.s.	(5.9)
	Ciriello *et al.*	2015	490	(100.0)	275	(56.1)	215	(43.9)	127	(100.0)	117	(92.1)	10	(7.9)
	Ellis *et al.*	2012	259	(100.0)	215	(83.0)	44	(17.0)	58	(100.0)	51	(87.9)	7	(12.1)

Supplemental Table 3 (Continued)

			NST						lobular BC					
			all cases		wt		mut		all cases		wt		mut	
		year	n	(%)	n	(%)	n	(%)	n	(%)	n	(%)	n	(%)
amplifications														
CCND1	Current study	2022	142	(100.0)	120	(84.5)	22	(15.5)	56	(100.0)	46	(82.1)	10	(17.9)
	Rinaldi *et al.*	2020	4896	(100.0)	4116	(84.1)	780	(15.9)	611	(100.0)	492	(80.5)	119	(19.5)
	Desmedt *et al.*	2016	300	(100.0)	n.s.	(63.7)	n.s.	(36.3)	145	(100.0)	n.s.	(66.9)	n.s.	(33.1)
	Ciriello *et al.*	2015	490	(100.0)	412	(84.1)	78	(15.9)	127	(100.0)	105	(82.7)	22	(17.3)
	Ellis *et al.*	2012	169	(100.0)	n.a.		n.a.		40	(100.0)	n.a.		n.a.	
FGFR1	Current study	2022	142	(100.0)	138	(97.2)	4	(2.8)	56	(100.0)	55	(98.2)	1	(1.8)
	Rinaldi *et al.*	2020	4896	(100.0)	4255	(86.9)	641	(13.1)	611	(100.0)	559	(91.5)	52	(8.5)
	Desmedt *et al.*	2016	300	(100.0)	n.s.	(61.0)	n.s.	(39.0)	145	(100.0)	n.s.	(74.5)	n.s.	(25.5)
	Ciriello *et al.*	2015	490	(100.0)	n.a.		n.a.		127	(100.0)	n.a.		n.a.	
	Ellis *et al.*	2012	169	(100.0)	n.a.		n.a.		40	(100.0)	n.a.		n.a.	
PAK1	Current study	2022	142	(100.0)	140	(98.6)	2	(1.4)	56	(100.0)	53	(94.6)	3	(5.4)
	Rinaldi *et al.*	2020	4896	(100.0)	n.a.		n.a.		611	(100.0)	n.a.		n.a.	
	Desmedt *et al.*	2016	300	(100.0)	n.s.	(80.3)	n.s.	(19.7)	145	(100.0)	n.s.	(80.7)	n.s.	(19.3)
	Ciriello *et al.*	2015	490	(100.0)	439	(89.6)	51	(10.4)	127	(100.0)	117	(92.1)	10	(7.9)
	Ellis *et al.*	2012	169	(100.0)	n.a.		n.a.		40	(100.0)	n.a.		n.a.	

Supplemental Table 4. Clinicopathological parameters associated with *ERBB2*-mutated BC.

Unless otherwise stated, the values are given in the format n (%), with n corresponding to the number of patients. The Fisher's Exact Test (FET) and Chi-Square Test for Trends (CSTT) were used for statistical analysis. Significant differences are highlighted in bold.

[a] Low expression (ER and PR status) is defined as 1-9% positive cells.

[b] Patients classified into low-high group were excluded for statistical analysis.

Abbreviations: mut, Patients with *ERBB2* mutation; wt, *ERBB2* wild-type; yrs, years; ER, estrogen receptor; pET, preoperative endocrine therapy; PR, progesterone receptor; RS, recurrence score; TAM, tamoxifen; AI, aromatase inhibitors

		all BC cases (n=622)							non-lobular BC (n=443)							lobular BC (n=179)								
		all cases		*ERBB2* wt		*ERBB2* mut		P-value	all cases		*ERBB2* wt		*ERBB2* mut		P-value	all cases		*ERBB2* wt		*ERBB2* mut		P-value		
		n	(%)	n	(%)	n	(%)		n	(%)	n	(%)	n	(%)		n	(%)	n	(%)	n	(%)		test	notes
all cases		622	(100.0)	604	(100.0)	18	(100.0)		443	(100.0)	439	(100.0)	4	(100.0)		179	(100.0)	165	(100.0)	14	(100.0)			
age at diagnosis	median (range) in yrs	54	(28-76)	54	(28-76)	55	(33-73)		54	(28-76)	54	(28-76)	48	(33-68)		57	(28-75)	57	(28-75)	55	(33-73)			
pT stage	pT1	371	(59.6)	359	(59.5)	12	(66.7)	.6317	274	(61.8)	271	(61.7)	3	(75.0)	1.0000	97	(54.2)	88	(53.3)	9	(64.3)	.5787	FET	pT1 vs pT2+
	pT2	223	(35.9)	217	(35.9)	6	(33.3)	1.0000	154	(34.8)	153	(34.9)	1	(25.0)	1.0000	69	(38.5)	64	(38.8)	5	(35.7)	.6031	FET	pT1/2 vs pT3+
	pT3	24	(3.9)	24	(4.0)	0	(0.0)		12	(2.7)	12	(2.7)	0	(0.0)		12	(6.7)	12	(7.3)	0	(0.0)			
	pT4	2	(0.3)	2	(0.3)	0	(0.0)		1	(0.2)	1	(0.2)	0	(0.0)		1	(0.6)	1	(0.6)	0	(0.0)			
	n.a.	2	(0.3)	2	(0.3)	0	(0.0)		2	(0.5)	2	(0.5)	0	(0.0)		0	(0.0)	0	(0.0)	0	(0.0)			
pN stage	pN0	541	(87.0)	523	(86.6)	18	(100.0)	.1492	379	(85.5)	375	(85.4)	4	(100.0)	1.0000	162	(90.5)	148	(89.7)	14	(100.0)	.3677	FET	pN0 vs pN1+
	pN1+	79	(12.7)	79	(13.1)	0	(0.0)		62	(14.0)	62	(14.1)	0	(0.0)		17	(9.5)	17	(10.3)	0	(0.0)			
	n.a.	2	(0.3)	2	(0.3)	0	(0.0)		2	(0.5)	2	(0.5)	0	(0.0)		0	(0.0)	0	(0.0)	0	(0.0)			
histological grade, baseline	G1	46	(7.4)	45	(7.5)	1	(5.6)	.4257	33	(7.4)	32	(7.3)	1	(25.0)	.3108	13	(7.3)	13	(7.9)	0	(0.0)	.7388	FET	G1/2 vs G3
	G2	399	(64.1)	385	(63.7)	14	(77.7)		268	(60.5)	265	(60.4)	3	(75.0)		131	(73.2)	120	(72.7)	11	(78.6)			
	G3	177	(28.5)	174	(28.8)	3	(16.7)		142	(32.1)	142	(32.3)	0	(0.0)		35	(19.5)	32	(19.4)	3	(21.4)			
	n.a.	0	(0.0)	0	(0.0)	0	(0.0)		0	(0.0)	0	(0.0)	0	(0.0)		0	(0.0)	0	(0.0)	0	(0.0)			
ER status, baseline	negative	1	(0.2)	1	(0.2)	0	(0.0)	1.0000	1	(0.2)	1	(0.2)	0	(0.0)	1.000	0	(0.0)	0	(0.0)	0	(0.0)	-	FET	ER pos vs neg
	low expression [a]	0	(0.0)	0	(0.0)	0	(0.0)		0	(0.0)	0	(0.0)	0	(0.0)		0	(0.0)	0	(0.0)	0	(0.0)			
	positive	620	(99.6)	602	(99.6)	18	(100.0)		442	(99.8)	438	(99.8)	4	(100.0)		178	(99.4)	164	(99.4)	14	(100.0)			
	n.a.	1	(0.2)	1	(0.2)	0	(0.0)		0	(0.0)	0	(0.0)	0	(0.0)		1	(0.6)	1	(0.6)	0	(0.0)			
ER status, post-pET	negative	0	(0.0)	0	(0.0)	0	(0.0)	-	0	(0.0)	0	(0.0)	0	(0.0)	-	0	(0.0)	0	(0.0)	0	(0.0)	-	FET	ER pos vs neg
	low expression [a]	1	(0.2)	0	(0.0)	1	(5.6)		0	(0.0)	0	(0.0)	0	(0.0)		1	(0.6)	0	(0.0)	1	(7.1)			
	positive	620	(99.6)	603	(99.8)	17	(94.4)		442	(99.8)	438	(99.8)	4	(100.0)		178	(99.4)	165	(100.0)	13	(92.9)			
	n.a.	1	(0.2)	1	(0.2)	0	(0.0)		1	(0.2)	1	(0.2)	0	(0.0)		0	(0.0)	0	(0.0)	0	(0.0)			
PR status, baseline	negative	46	(7.4)	45	(7.5)	1	(5.6)	1.0000	32	(7.2)	32	(7.3)	0	(0.0)	1.0000	14	(7.8)	13	(7.9)	1	(7.1)	1.0000	FET	PR pos vs neg
	low expression [a]	27	(4.3)	26	(4.3)	1	(5.6)		22	(5.0)	22	(5.0)	0	(0.0)		5	(2.8)	4	(2.4)	1	(7.1)			
	positive	549	(88.3)	533	(88.2)	16	(88.8)		389	(87.8)	385	(87.7)	4	(100.0)		160	(89.4)	148	(89.7)	12	(85.8)			
	n.a.	0	(0.0)	0	(0.0)	0	(0.0)		0	(0.0)	0	(0.0)	0	(0.0)		0	(0.0)	0	(0.0)	0	(0.0)			
PR status, post-pET	negative	137	(22.0)	132	(21.8)	5	(27.8)	.5651	99	(22.4)	98	(22.3)	1	(25.0)	1.0000	38	(21.2)	34	(20.6)	4	(28.6)	.4999	FET	PR pos vs neg
	low expression [a]	65	(10.5)	65	(10.8)	0	(0.0)		48	(10.8)	48	(10.9)	0	(0.0)		17	(9.5)	17	(10.3)	0	(0.0)			
	positive	420	(67.5)	407	(67.4)	13	(72.2)		296	(66.8)	293	(66.8)	3	(75.0)		124	(69.3)	114	(69.1)	10	(71.4)			
	n.a.	0	(0.0)	0	(0.0)	0	(0.0)		0	(0.0)	0	(0.0)	0	(0.0)		0	(0.0)	0	(0.0)	0	(0.0)			
HER2 status, baseline (according to ASCO/CAP guidelines)	negative	615	(98.9)	598	(99.0)	17	(94.4)	1.0000	437	(98.7)	433	(98.7)	4	(100.0)	1.0000	178	(99.4)	165	(100.0)	13	(92.9)	-	FET	HER2 pos vs neg
	positive	5	(0.8)	5	(0.8)	0	(0.0)		5	(1.1)	5	(1.1)	0	(0.0)		0	(0.0)	0	(0.0)	0	(0.0)			
	n.a.	2	(0.3)	1	(0.2)	1	(5.6)		1	(0.2)	1	(0.2)	0	(0.0)		1	(0.6)	0	(0.0)	1	(7.1)			
HER2 status, post-pET (according to ASCO/CAP guidelines)	negative	613	(98.5)	596	(98.6)	17	(94.4)	.2104	435	(98.2)	431	(98.2)	4	(100.0)	1.0000	178	(99.4)	165	(100.0)	13	(92.9)	.0782	FET	HER2 pos vs neg
	positive	8	(1.3)	7	(1.2)	1	(5.6)		7	(1.6)	7	(1.6)	0	(0.0)		1	(0.6)	0	(0.0)	1	(7.1)			
	n.a.	1	(0.2)	1	(0.2)	0	(0.0)		1	(0.2)	1	(0.2)	0	(0.0)		0	(0.0)	0	(0.0)	0	(0.0)			

Supplemental Table 4 (Continued)

		all BC cases (n=622)							non-lobular BC (n=443)							lobular BC (n=179)								
		**all cases**		***ERBB2* wt**		***ERBB2* mut**			**all cases**		***ERBB2* wt**		***ERBB2* mut**			**all cases**		***ERBB2* wt**		***ERBB2* mut**				
		n	(%)	n	(%)	n	(%)	P-value	n	(%)	n	(%)	n	(%)	P-value	n	(%)	n	(%)	n	(%)	P-value	test	notes
Ki67,	0-9	72	(11.6)	70	(11.6)	2	(11.1)	1.0000	42	(9.5)	41	(9.3)	1	(25.0)	.3296	30	(16.8)	29	(17.6)	1	(7.1)	.4704	FET	0-9 vs 10-100
baseline	10-19	244	(39.2)	234	(38.7)	10	(55.6)		163	(36.8)	162	(36.9)	1	(25.0)		81	(45.2)	72	(43.6)	9	(64.3)			
	20-34	222	(35.7)	217	(35.9)	5	(27.7)		171	(38.6)	169	(38.5)	2	(50.0)		51	(28.5)	48	(29.1)	3	(21.5)			
	35-100	84	(13.5)	83	(13.8)	1	(5.6)		67	(15.1)	67	(15.3)	0	(0.0)		17	(9.5)	16	(9.7)	1	(7.1)			
	n.a.	0	(0.0)	0	(0.0)	0	(0.0)		0	(0.0)	0	(0.0)	0	(0.0)		0	(0.0)	0	(0.0)	0	(0.0)			
Ki67,	0-9	327	(52.6)	321	(53.1)	6	(33.3)	.1489	223	(50.4)	221	(50.4)	2	(50.0)	1.0000	104	(58.1)	100	(60.6)	4	(28.6)	**.0248**	FET	0-9 vs 10-100
post-pET	10-19	186	(29.9)	176	(29.1)	10	(55.6)		134	(30.2)	133	(30.3)	1	(25.0)		52	(29.0)	43	(26.1)	9	(64.3)			
	20-34	87	(14.0)	85	(14.1)	2	(11.1)		67	(15.1)	66	(15.0)	1	(25.0)		20	(11.2)	19	(11.5)	1	(7.1)			
	35-100	22	(3.5)	22	(3.7)	0	(0.0)		19	(4.3)	19	(4.3)	0	(0.0)		3	(1.7)	3	(1.8)	0	(0.0)			
	n.a.	0	(0.0)	0	(0.0)	0	(0.0)		0	(0.0)	0	(0.0)	0	(0.0)		0	(0.0)	0	(0.0)	0	(0.0)			
Ki67,	low-low	59	(9.5)	57	(9.4)	2	(11.1)	.2114	38	(8.6)	37	(8.4)	1	(25.0)	.6274	21	(11.7)	20	(12.1)	1	(7.1)	**.0263**	CSTT	
dynamic categories [b]	high-low	268	(43.1)	264	(43.7)	4	(22.2)	.0947	185	(41.8)	184	(41.9)	1	(25.0)	1.0000	83	(46.4)	80	(48.5)	3	(21.4)	**.0187**	FET	high/high vs x/low
	high-high	282	(45.3)	270	(44.7)	12	(66.7)		216	(48.7)	214	(48.8)	2	(50.0)		66	(36.9)	56	(33.9)	10	(71.5)			
	low-high	13	(2.1)	13	(2.2)	0	(0.0)		4	(0.9)	4	(0.9)	0	(0.0)		9	(5.0)	9	(5.5)	0	(0.0)			
	n.a.	0	(0.0)	0	(0.0)	0	(0.0)		0	(0.0)	0	(0.0)	0	(0.0)		0	(0.0)	0	(0.0)	0	(0.0)			
E-cadherin,	negative	171	(27.5)	159	(26.3)	12	(66.7)	**.0002**	11	(2.5)	11	(2.5)	0	(0.0)	1.0000	160	(89.4)	148	(89.7)	12	(85.7)	1.0000	FET	E-cad pos vs neg
baseline	positive	411	(66.1)	407	(67.4)	4	(22.2)		402	(90.7)	398	(90.7)	4	(100.0)		9	(5.0)	9	(5.5)	0	(0.0)			
	n.a.	40	(6.4)	38	(6.3)	2	(11.1)		30	(6.8)	30	(6.8)	0	(0.0)		10	(5.6)	8	(4.8)	2	(14.3)			
E-cadherin,	negative	169	(27.2)	155	(25.7)	14	(77.8)	**<.0001**	9	(2.0)	9	(2.1)	0	(0.0)	1.0000	160	(89.4)	146	(88.5)	14	(100.0)	1.0000	FET	E-cad pos vs neg
post-pET	positive	418	(67.2)	414	(68.5)	4	(22.2)		408	(92.1)	404	(92.0)	4	(100.0)		10	(5.6)	10	(6.0)	0	(0.0)			
	n.a.	35	(5.6)	35	(5.8)	0	(0.0)		26	(5.9)	26	(5.9)	0	(0.0)		9	(5.0)	9	(5.5)	0	(0.0)			
Oncotype DX RS,	0-11	142	(22.8)	139	(23.0)	3	(16.7)	.2929	101	(22.8)	101	(22.8)	0	(0.0)	1.0000	41	(22.9)	38	(23.0)	3	(21.4)	**.0087**	FET	RS 0-25 vs 26-100
baseline	12-25	362	(58.2)	354	(58.6)	8	(44.4)		241	(54.4)	238	(54.4)	3	(75.0)		121	(67.6)	116	(70.3)	5	(35.7)			
	26-100	101	(16.3)	97	(16.1)	4	(22.2)		87	(19.6)	87	(19.6)	0	(0.0)		14	(7.8)	10	(6.1)	4	(28.6)			
	n.a.	17	(2.7)	14	(2.3)	3	(16.7)		14	(3.2)	13	(3.2)	1	(25.0)		3	(1.7)	1	(0.6)	2	(14.3)			
pET agent	tamoxifen	286	(46.0)	279	(46.2)	7	(38.9)	.6346	204	(46.0)	202	(46.0)	2	(50.0)	1.0000	82	(45.8)	77	(46.7)	5	(35.7)	.5787	FET	TAM vs AI
	aromatase inhibitors	334	(53.7)	323	(53.5)	11	(61.1)		237	(53.5)	235	(53.5)	2	(50.0)		97	(54.2)	88	(53.3)	9	(64.3)			
	n.a.	2	(0.3)	2	(0.3)	0	(0.0)		2	(0.5)	2	(0.5)	0	(0.0)		0	(0.0)	0	(0.0)	0	(0.0)			

Supplemental Table 5. Genetic alterations and their distribution according to post-pET Ki67.

The values are given in the format n (%), with n corresponding to the number of patients. The Fisher's Exact Test (post-pET Ki67 >10% vs ≤10%) was used for statistical analysis. Significant values are highlighted in bold.

Abbreviations: mut, mutations in the gene under consideration; wt, wild-type for the gene under consideration; amp, amplification of the gene under consideration; n-amp, no amplification of the gene under consideration; TAM-cohort, cases treated with tamoxifen; AI-cohort, cases treated with aromatase inhibitors

**post-pET Ki67**		**all BC cases (n=622)**									**non-lobular BC (n=443)**									**lobular BC (n=179)**								
		0-9%		10-19%		20-34%		35-100%		P-value	0-9%		10-19%		20-34%		35-100%		P-value	0-9%		10-19%		20-34%		35-100%		P-value
association with mutations																												
ARID1A	mut	15	(4.6)	13	(7.0)	4	.(4.6)	1	(4.5)	.4747	8	(3.6)	10	(7.5)	3	(4.5)	1	(5.3)	.1960	7	(6.7)	3	(5.8)	1	(5.0)	0	(0.0)	.7634
	wt	312	(95.4)	173	(93.0)	83	(95.4)	21	(95.5)		215	(96.4)	124	(92.5)	64	(95.5)	18	(94.7)		97	(93.3)	49	(94.2)	19	(95.0)	3	(100.0)	
CDH1	mut	77	(23.6)	45	(24.2)	13	(14.9)	0	(0.0)	.5560	3	(1.4)	6	(4.5)	2	(3.0)	0	(0.0)	.1388	74	(71.2)	39	(75.0)	11	(55.0)	0	(0.0)	.6226
	wt	250	(76.4)	141	(75.8)	74	(85.1)	22	(100.0)		220	(68.6)	128	(95.5)	65	(97.0)	19	(100.0)		30	(28.1)	13	(25.0)	9	(45.0)	3	(100.0)	
ERBB2	mut	6	(1.8)	10	(5.4)	2	(2.3)	0	(0.0)	.1489	2	(0.9)	1	(0.8)	1	(1.5)	0	(0.0)	1.0000	4	(3.9)	9	(17.3)	1	(5.0)	0	(0.0)	**.0248**
	wt	321	(98.2)	176	(94.6)	85	(97.7)	22	(100.0)		221	(99.1)	133	(99.3)	66	(98.5)	19	(100.0)		100	(96.1)	43	(82.7)	19	(95.0)	3	(100.0)	
GATA3	mut	55	(16.8)	31	(16.7)	14	(16.1)	3	(13.6)	.9141	49	(22.0)	27	(20.2)	13	(19.4)	3	(15.8)	.5593	6	(5.8)	4	(7.7)	1	(5.0)	0	(0.0)	1.0000
	wt	272	(83.2)	155	(83.3)	73	(83.9)	19	(86.4)		174	(78.0)	107	(79.8)	54	(80.6)	16	(84.2)		98	(94.2)	48	(92.3)	19	(95.0)	3	(0.0)	
PIK3CA	mut	152	(46.5)	73	(39.2)	32	(36.8)	12	(54.5)	.0894	103	(46.2)	57	(42.5)	23	(34.3)	11	(57.9)	.3385	49	(47.1)	16	(30.8)	9	(45.0)	1	(33.3)	.1246
	wt	175	(53.5)	113	(60.8)	55	(63.2)	10	(45.5)		120	(53.8)	77	(57.5)	44	(65.7)	8	(42.1)		55	(52.9)	36	(69.2)	11	(55.0)	2	(66.7)	
TP53	mut	16	(4.9)	19	(10.2)	16	(18.4)	5	(22.7)	**.0002**	14	(6.3)	16	(11.9)	14	(20.9)	5	(26.3)	**.0014**	2	(1.9)	3	(5.8)	2	(10.0)	0	(0.0)	.1317
	wt	311	(95.1)	167	(89.8)	71	(81.6)	17	(77.3)		209	(93.7)	118	(88.1)	53	(79.1)	14	(73.7)		102	(98.1)	49	(94.2)	18	(90.0)	3	(100.0)	
association with amplifications																												
CCND1	amp	18	(17.8)	7	(12.3)	6	(19.4)	1	(10.0)	.5649	9	(13.4)	6	(14.3)	6	(23.1)	1	(12.5)	.6447	9	(26.5)	1	(6.7)	0	(0.0)	0	(0.0)	.0703
	n-amp	83	(82.2)	50	(87.7)	25	(80.6)	9	(90.0)		58	(86.6)	36	(87.7)	20	(76.9)	7	(87.5)		25	(73.5)	14	(93.3)	5	(100.0)	2	(100.0)	
FGFR1	amp	3	(3.0)	1	(1.8)	1	(3.2)	1	(10.0)	1.0000	2	(3.0)	1	(2.4)	1	(3.9)	1	(12.5)	1.0000	1	(2.9)	0	(0.0)	0	(0.0)	0	(0.0)	1.0000
	n-amp	98	(97.0)	56	(98.2)	30	(96.8)	9	(90.0)		65	(97.0)	41	(97.6)	25	(96.1)	7	(87.5)		33	(97.1)	15	(100.0)	5	(100.0)	2	(100.0)	
PAK1	amp	4	(4.0)	1	(1.8)	0	(0.0)	0	(0.0)	.3689	1	(1.5)	1	(2.4)	0	(0.0)	0	(0.0)	1.0000	3	(8.8)	0	(0.0)	0	(0.0)	0	(0.0)	.2714
	n-amp	97	(96.0)	56	(98.2)	31	(100.0)	10	(100.0)		66	(98.5)	41	(97.6)	26	(100.0)	8	(100.0)		31	(91.2)	15	(100.0)	5	(100.0)	2	(100.0)	

Supplemental Table 5 (Continued)

post-pET Ki67		all BC (TAM-cohort, n=286)									non-lobular BC (TAM-cohort, n=204)									lobular BC (TAM-cohort, n=82)								
		0-9%		10-19%		20-34%		35-100%		P-value	0-9%		10-19%		20-34%		35-100%		P-value	0-9%		10-19%		20-34%		35-100%		P-value
association with mutations																												
ARID1A	mut	6	(7.0)	6	(5.0)	3	(5.0)	1	(5.3)	.5762	2	(3.6)	6	(6.8)	2	(4.6)	1	(5.9)	.7307	4	(12.9)	0	(0.0)	1	(6.3)	0	(0.0)	.0650
	wt	80	(93.0)	115	(95.0)	57	(95.0)	18	(94.7)		53	(96.4)	82	(93.2)	42	(95.4)	16	(94.1)		27	(87.1)	33	(100.0)	15	(93.7)	2	(100.0)	
CDH1	mut	19	(22.1)	30	(24.8)	10	(16.7)	0	(0.0)	1.0000	1	(1.8)	4	(4.6)	1	(2.3)	0	(0.0)	1.0000	18	(58.1)	26	(78.8)	9	(56.3)	0	(0.0)	.3512
	wt	67	(77.9)	91	(75.2)	50	(83.3)	19	(100.0)		54	(98.2)	84	(95.4)	43	(97.7)	17	(100.0)		13	(41.9)	7	(21.2)	7	(43.7)	2	(100.0)	
ERBB2	mut	0	(0.0)	5	(4.1)	2	(3.3)	0	(0.0)	.1067	0	(0.0)	1	(1.1)	1	(2.3)	0	(0.0)	1.0000	0	(0.0)	4	(12.1)	1	(6.3)	0	(0.0)	.1511
	wt	86	(100.0)	116	(95.9)	58	(96.7)	19	(100.0)		55	(100.0)	87	(98.9)	43	(97.7)	17	(100.0)		31	(100.0)	29	(87.9)	15	(93.7)	2	(100.0)	
GATA3	mut	23	(26.7)	25	(20.7)	10	(16.7)	2	(10.5)	.1534	19	(34.6)	22	(25.0)	9	(20.5)	2	(11.8)	.1022	4	(12.9)	3	(9.1)	1	(6.3)	0	(0.0)	.4688
	wt	63	(73.3)	96	(79.3)	50	(83.3)	17	(89.5)		36	(65.5)	66	(75.0)	35	(79.5)	15	(88.2)		27	(87.1)	30	(90.9)	15	(93.7)	2	(100.0)	
PIK3CA	mut	40	(46.5)	49	(40.5)	22	(36.7)	12	(63.2)	.4380	29	(52.7)	37	(42.1)	15	(34.1)	11	(64.7)	.2064	11	(35.5)	12	(36.4)	7	(43.8)	1	(50.0)	.8165
	wt	46	(53.5)	72	(59.5)	38	(63.3)	7	(36.8)		26	(47.3)	51	(57.9)	29	(65.9)	6	(35.3)		20	(64.5)	21	(63.6)	9	(56.2)	1	(50.0)	
TP53	mut	3	(3.5)	8	(6.6)	11	(18.3)	4	(21.1)	**.0413**	2	(3.6)	6	(6.8)	10	(22.7)	4	(23.5)	.0716	1	(3.2)	2	(6.1)	1	(6.3)	0	(0.0)	1.0000
	wt	83	(96.5)	113	(93.4)	49	(81.7)	15	(78.9)		53	(96.4)	82	(93.2)	34	(77.3)	13	(76.5)		30	(96.8)	31	(93.9)	15	(93.7)	2	(100.0)	
association with amplifications																												
CCND1	amp	1	(5.3)	3	(8.6)	6	(24.0)	1	(11.1)	.4441	1	(7.7)	3	(12.0)	6	(30.0)	1	(12.5)	.6782	0	(0.0)	0	(0.0)	0	(0.0)	0	(0.0)	-
	n-amp	18	(94.7)	32	(9.4)	19	(76.0)	8	(88.9)		12	(92.3)	22	(88.0)	14	(70.0)	7	(87.5)		6	(100.0)	10	(100.0)	5	(100.0)	1	(100.0)	
FGFR1	amp	2	(10.5)	1	(2.9)	1	(4.0)	1	(11.1)	.2938	1	(7.7)	1	(4.0)	1	(5.0)	1	(12.5)	1.0000	1	(16.7)	0	(0.0)	0	(0.0)	0	(0.0)	.2727
	n-amp	17	(89.5)	34	(97.1)	24	(96.0)	8	(88.9)		12	(92.3)	24	(96.0)	19	(95.0)	7	(87.5)		5	(83.3)	10	(100.0)	5	(100.0)	1	(100.0)	
PAK1	amp	0	(0.0)	1	(2.9)	0	(0.0)	0	(0.0)	1.0000	0	(0.0)	1	(4.0)	0	(0.0)	0	(0.0)	1.0000	0	(0.0)	0	(0.0)	0	(0.0)	0	(0.0)	-
	n-amp	19	(100.0)	34	(97.1)	25	(100.0)	9	(100.0)		13	(100.0)	24	(96.0)	20	(100.0)	8	(100.0)		6	(100.0)	10	(100.0)	5	(100.0)	1	(100.0)	

post-pET Ki67		all BC (AI-cohort, n=334)									non-lobular BC (AI-cohort, n=237)									lobular BC (AI-cohort, n=97)								
		0-9%		10-19%		20-34%		35-100%		P-value	0-9%		10-19%		20-34%		35-100%		P-value	0-9%		10-19%		20-34%		35-100%		P-value
association with mutations																												
ARID1A	mut	9	(3.3)	7	(11.1)	1	(3.7)	0	(0.0)	.0931	6	(3.6)	4	(9.1)	1	(4.4)	0	(0.0)	.3056	3	(4.1)	3	(15.8)	0	(0.0)	0	(0.0)	.1589
	wt	232	(96.7)	56	(88.9)	26	(96.3)	3	(100.0)		162	(96.4)	40	(90.9)	22	(95.6)	2	(100.0)		70	(95.9)	16	(84.2)	4	(100.0)	1	(100.0)	
CDH1	mut	58	(24.1)	15	(23.8)	3	(11.1)	0	(0.0)	.3860	2	(1.2)	2	(4.6)	1	(4.4)	0	(0.0)	.1493	56	(76.7)	13	(68.4)	2	(50.0)	0	(0.0)	.1915
	wt	183	(75.9)	48	(76.2)	24	(77.9)	3	(100.0)		166	(98.8)	42	(95.4)	22	(95.6)	2	(100.0)		17	(23.3)	6	(31.6)	2	(50.0)	1	(100.0)	
ERBB2	mut	6	(2.5)	5	(7.9)	0	(0.0)	0	(0.0)	.0543	2	(1.2)	0	(0.0)	0	(0.0)	0	(0.0)	1.0000	4	(5.5)	5	(26.3)	0	(0.0)	0	(0.0)	**.0389**
	wt	235	(97.5)	58	(92.1)	27	(100.0)	3	(100.0)		166	(98.8)	44	(100.0)	23	(100.0)	2	(100.0)		69	(94.5)	14	(73.7)	4	(100.0)	1	(100.0)	
GATA3	mut	32	(13.3)	6	(9.5)	4	(14.8)	1	(33.3)	.8558	30	(17.9)	5	(11.4)	4	(17.4)	1	(50.0)	.5734	2	(2.7)	1	(5.3)	0	(0.0)	0	(0.0)	1.0000
	wt	209	(86.7)	57	(90.5)	23	(85.2)	2	(66.7)		138	(82.1)	39	(88.6)	19	(82.6)	1	(50.0)		71	(97.3)	18	(94.7)	4	(100.0)	1	(100.0)	
PIK3CA	mut	112	(46.5)	24	(38.1)	10	(37.0)	0	(0.0)	.1107	74	(44.1)	20	(45.5)	8	(34.8)	0	(0.0)	.6665	38	(52.1)	4	(21.1)	2	(50.0)	0	(0.0)	**.0323**
	wt	129	(53.5)	39	(61.9)	17	(63.0)	3	(100.0)		94	(55.9)	24	(54.5)	15	(65.2)	2	(100.0)		35	(47.9)	15	(78.9)	2	(50.0)	1	(100.0)	
TP53	mut	13	(5.4)	10	(15.9)	5	(18.5)	1	(33.3)	**.0017**	12	(7.1)	9	(20.5)	4	(17.4)	1	(50.0)	**.0054**	1	(1.4)	1	(5.3)	1	(25.0)	0	(0.0)	.1504
	wt	228	(94.6)	53	(84.1)	22	(81.5)	2	(66.7)		156	(92.9)	35	(79.5)	19	(82.6)	1	(50.0)		72	(98.6)	18	(94.7)	3	(75.0)	1	(100.0)	
association with amplifications																												
CCND1	amp	17	(20.7)	4	(18.2)	0	(0.0)	0	(0.0)	.5827	8	(14.8)	3	(17.7)	0	(0.0)	0	-	1.0000	9	(32.1)	1	(20.0)	0	-	0	(0.0)	.6445
	n-amp	65	(79.3)	18	(81.8)	6	(100.0)	1	(100.0)		46	(85.2)	14	(82.3)	6	(100.0)	0	-		19	(67.9)	4	(80.0)	0	-	1	(100.0)	
FGFR1	amp	1	(1.2)	0	(0.0)	0	(0.0)	0	(0.0)	1.0000	1	(1.9)	0	(0.0)	0	(0.0)	0	-	1.0000	0	(0.0)	0	(0.0)	0	(0.0)	0	(0.0)	-
	n-amp	81	(98.8)	22	(100.0)	6	(100.0)	1	(100.0)		53	(98.1)	17	(100.0)	6	(100.0)	0	-		28	(100.0)	5	(100.0)	0	(100.0)	1	(100.0)	
PAK1	amp	4	(4.9)	0	(0.0)	0	(0.0)	0	(0.0)	.5712	1	(1.9)	0	(0.0)	0	(0.0)	0	-	1.0000	3	(10.7)	0	(0.0)	0	-	0	(0.0)	1.0000
	n-amp	78	(95.1)	22	(100.0)	6	(100.0)	1	(100.0)		53	(98.1)	17	(100.0)	6	(100.0)	0	-		25	(89.3)	5	(100.0)	0	-	1	(100.0)	

Supplemental Table 6. Genetic alterations and their distribution according to dynamic Ki67 categories.

The values are given in the format n (%), with n corresponding to the number of patients. According to Ki67 indices patients/BCs were classified four categories: (i) low-low (baseline and post-pET Ki67 <10%, light grey), (ii) high-low (baseline Ki67 ≥10%, and post-pET Ki67 <10%, grey), (iii) high-high (baseline and post-pET Ki67 both ≥10%, dark grey), and low-high (baseline Ki67 <10%, and post-pET Ki67 ≥10%, data not shown because of the low number cases in this category, as indicated in Table 1). This this categorization is consistent with similar analyses in the POETIC trial (Smith *et al.* 2020). The Chi-Square Test for Trends (CSTT) was used for statistical analysis. The low-high category was excluded, because of the low number of patients. Significant differences are highlighted in bold.

<u>Abbreviations:</u> mut, mutations in the gene under consideration; wt, wild-type for the gene under consideration; amp, amplification of the gene under consideration; n-amp, no amplification of the gene under consideration

**post-pET Ki67**		**all BC cases (n=622)**					**non-lobular BC (n=443)**					**lobular BC (n=179)**				
		low-low	high-low	high-high	low-high	P-value	low-low	high-low	high-high	low-high	P-value	low-low	high-low	high-high	low-high	P-value
association with mutations																
ARID1A	mut	3 (5.1)	12 (4.5)	17 (6.0)	1 (7.7)	.5253	2 (5.3)	6 (3.2)	14 (6.5)	0 (0.0)	.2952	1 (4.8)	6 (7.2)	3 (4.5)	1 (11.1)	.7510
	wt	56 (94.9)	256 (95.5)	265 (94.0)	12 (92.3)		36 (94.7)	179 (96.8)	202 (93.5)	4 (100.0)		20 (95.2)	77 (92.8)	63 (95.5)	8 (88.9)	
CDH1	mut	18 (30.5)	59 (22.0)	54 (19.1)	4 (30.8)	.0705	0 (0.0)	3 (1.6)	8 (3.7)	0 (0.0)	.0930	18 (85.7)	56 (67.5)	46 (69.7)	4 (44.4)	.3403
	wt	41 (69.5)	209 (78.0)	228 (80.9)	9 (69.2)		38 (100.0)	182 (98.4)	208 (96.3)	4 (100.0)		3 (14.3)	27 (32.5)	20 (30.3)	5 (55.6)	
ERBB2	mut	2 (3.4)	4 (1.5)	12 (4.3)	0 (0.0)	.2114	1 (2.6)	1 (0.5)	2 (0.9)	0 (0.0)	.6274	1 (4.8)	3 (3.6)	10 (15.2)	0 (0.0)	**.0263**
	wt	57 (96.6)	264 (98.5)	270 (95.7)	13 (100.0)		37 (97.4)	184 (99.5)	214 (99.1)	4 (100.0)		20 (95.2)	80 (96.4)	56 (84.8)	9 (100.0)	
GATA3	mut	13 (22.0)	42 (15.7)	48 (17.0)	0 (0.0)	.6528	12 (31.6)	37 (20.0)	43 (19.9)	0 (0.0)	.2508	1 (4.8)	5 (6.0)	5 (7.6)	0 (0.0)	.6097
	wt	46 (78.0)	226 (84.3)	234 (83.0)	13 (100.0)		26 (68.4)	148 (80.0)	173 (80.1)	4 (100.0)		20 (95.2)	78 (94.0)	61 (92.4)	9 (100.0)	
PIK3CA	mut	30 (50.8)	122 (45.5)	112 (39.7)	5 (38.5)	.0660	23 (60.5)	80 (43.2)	88 (40.7)	3 (75.0)	.0627	7 (33.3)	42 (50.6)	24 (36.4)	2 (22.2)	.5880
	wt	29 (49.2)	146 (54.5)	170 (60.3)	8 (61.5)		15 (39.5)	105 (56.8)	128 (59.3)	1 (25.0)		14 (66.7)	41 (49.4)	42 (63.6)	7 (77.8)	
TP53	mut	2 (3.4)	14 (5.2)	40 (14.2)	0 (0.0)	**.0002**	1 (2.6)	13 (7.0)	35 (16.2)	0 (0.0)	**.0009**	1 (4.8)	1 (1.2)	5 (7.6)	0 (0.0)	.2124
	wt	57 (96.6)	254 (94.8)	242 (85.8)	13 (100.0)		37 (97.4)	172 (93.0)	181 (83.8)	4 (100.0)		20 (95.2)	82 (98.8)	61 (92.4)	9 (100.0)	
association with amplifications																
CCND1	amp	1 (9.1)	17 (18.9)	14 (15.1)	0 (0.0)	.8649	1 (14.3)	8 (13.3)	13 (17.6)	0 (0.0)	.5434	0 (0.0)	9 (30.0)	1 (5.3)	0 (0.0)	.2801
	n-amp	10 (90.9)	73 (81.1)	79 (84.9)	5 (100.0)		6 (85.7)	52 (86.7)	61 (82.4)	2 (100.0)		4 (100.0)	21 (70.0)	18 (94.7)	3 (100.0)	
FGFR1	amp	0 (0.0)	3 (3.3)	3 (3.2)	0 (0.0)	.7476	0 (0.0)	2 (3.3)	3 (4.1)	0 (0.0)	.6303	0 (0.0)	1 (3.3)	0 (0.0)	0 (0.0)	.6103
	n-amp	11 (100.0)	87 (96.7)	90 (96.8)	5 (100.0)		7 (100.0)	58 (96.7)	71 (95.9)	2 (100.0)		4 (100.0)	29 (96.7)	19 (100.0)	3 (100.0)	
PAK1	amp	0 (0.0)	4 (4.4)	1 (1.1)	0 (0.0)	.3988	0 (0.0)	1 (1.7)	1 (1.4)	0 (0.0)	.9523	0 (0.0)	3 (10.0)	0 (0.0)	0 (0.0)	.3962
	n-amp	11 (100.0)	86 (95.6)	92 (98.9)	5 (100.0)		7 (100.0)	59 (98.3)	73 (98.6)	2 (100.0)		4 (100.0)	27 (90.0)	19 (100.0)	3 (100.0)	
